# VGLL3 operates via TEAD1, TEAD3 and TEAD4 to influence myogenesis in skeletal muscle

**DOI:** 10.1242/jcs.225946

**Published:** 2019-07-05

**Authors:** Nicolas Figeac, Abdalla D. Mohamed, Congshan Sun, Martin Schönfelder, David Matallanas, Amaya Garcia-Munoz, Edoardo Missiaglia, Elaina Collie-Duguid, Vanessa De Mello, Ajaybabu V. Pobbati, Johanna Pruller, Oihane Jaka, Stephen D. R. Harridge, Wanjin Hong, Janet Shipley, Neil Vargesson, Peter S. Zammit, Henning Wackerhage

**Affiliations:** 1 Randall Centre for Cell and Molecular Biophysics, King's College London, London SE1 1UL, UK; 2 School of Medicine, Medical Sciences and Nutrition, University of Aberdeen, Foresterhill, Aberdeen AB25 2ZD, UK; 3 Institute of Developmental Genetics, Helmholtz Zentrum München, German Research Center for Environment and Health, Ingolstaedter Landstrasse 1, D-85764 Munich/Neuherberg, Germany; 4 Department of Neurology, The Johns Hopkins School of Medicine, Baltimore, MD 21205, USA; 5 Faculty of Sport and Health Sciences, Technical University of Munich, Georg-Brauchle-Ring 60, 80992 Munich, Germany; 6 Systems Biology Ireland, Conway Institute, Belfield; Dublin 4, Ireland; 7 Institute of Pathology, Lausanne University Hospital (CHUV), 1011 Lausanne, Switzerland; 8 University of Aberdeen, Centre for Genome Enabled Biology and Medicine, 23 St Machar Drive, Aberdeen AB24 3RY, UK; 9 Institute of Molecular and Cell Biology, A-STAR, 61 Biopolis Drive, Singapore 138673, Singapore; 10 Centre for Human and Applied Physiological Sciences, King's College London, London SE1 1UL, UK; 11 Sarcoma Molecular Pathology Team, Divisions of Molecular Pathology and Cancer Therapeutics, Institute of Cancer Research, Surrey, SM2 5NG, UK

**Keywords:** VGLL3, TEAD, YAP, TAZ, WWTR1, Skeletal muscle, Stem cells

## Abstract

VGLL proteins are transcriptional co-factors that bind TEAD family transcription factors to regulate events ranging from wing development in fly, to muscle fibre composition and immune function in mice. Here, we characterise *Vgll3* in skeletal muscle. We found that mouse *Vgll3* was expressed at low levels in healthy muscle but that its levels increased during hypertrophy or regeneration; in humans, *VGLL3* was highly expressed in tissues from patients with various muscle diseases, such as in dystrophic muscle and alveolar rhabdomyosarcoma. Interaction proteomics revealed that VGLL3 bound TEAD1, TEAD3 and TEAD4 in myoblasts and/or myotubes. However, there was no interaction with proteins from major regulatory systems such as the Hippo kinase cascade, unlike what is found for the TEAD co-factors YAP (encoded by *YAP1*) and TAZ (encoded by *WWTR1*). *Vgll3* overexpression reduced the activity of the Hippo negative-feedback loop, affecting expression of muscle-regulating genes including *Myf5*, *Pitx2* and *Pitx3*, and genes encoding certain Wnts and IGFBPs. VGLL3 mainly repressed gene expression, regulating similar genes to those regulated by YAP and TAZ. siRNA-mediated *Vgll3* knockdown suppressed myoblast proliferation, whereas *Vgll3* overexpression strongly promoted myogenic differentiation. However, skeletal muscle was overtly normal in *Vgll3*-null mice, presumably due to feedback signalling and/or redundancy. This work identifies VGLL3 as a transcriptional co-factor operating with the Hippo signal transduction network to control myogenesis.

## Abstract (precis)

**Summary:** VGLL3 interacts with TEAD transcription factors to direct expression of crucial muscle regulatory genes and contribute to the control of skeletal myogenesis.

## INTRODUCTION

The Hippo signal transduction network regulates development, stem cell identity and function, cell proliferation, and organ and body size (Hansen et al., 2015; Piccolo et al., 2014; Tremblay and Camargo, 2012). Hippo proteins also have major functions in skeletal muscle (Wackerhage et al., 2014), where the Hippo effector YAP (gene symbol *YAP1*), but also other proteins such as the YAP paralogue TAZ (gene symbol *WWTR1*), vestigial-like factors (VGLL) 1–4 and the TEA domain (TEAD) 1–4 transcription factors, have been linked to both developmental and regenerative myogenesis (Chen et al., 2004; Gunther et al., 2004; Judson et al., 2012; Maeda et al., 2002; Mielcarek et al., 2002, 2009; Sun et al., 2017; Watt et al., 2010). For example, both YAP and TAZ are expressed in muscle stem (satellite) cells, where they promote proliferation, and while TAZ enhances subsequent myogenic differentiation into multinucleated myotubes, YAP inhibits this process (Judson et al., 2012; Sun et al., 2017). This transcriptional network also plays a role in genetic muscle disease (Bertrand et al., 2014; Judson et al., 2013) and development of rhabdomyosarcomas – sarcomas with characteristics of skeletal muscle (Mohamed et al., 2016; Slemmons et al., 2015; Tremblay et al., 2014). Hippo and VGLL proteins also affect muscle fibre type distribution (Honda et al., 2017; Tsika et al., 2008), and can cause skeletal muscle hypertrophy (Goodman et al., 2015; Watt et al., 2015). Finally, Hippo proteins are candidate regulators of adaptation to exercise training (Gabriel et al., 2016).

The term ‘Hippo’ stems from a kinase-encoding gene whose mutagenesis resulted in the puckered appearance of the head in fly, reminiscent of the skin of a hippopotamus. At the centre of the Hippo signal transduction network are the transcriptional co-factors YAP and TAZ that bind and co-activate the TEAD1–TEAD4 transcription factors to regulate gene expression. The Hippo pathway is a signalling cascade where the kinases MST1 and MST2 (encoded by *STK4* and *STK3*, respectively) regulate LATS1 and LATS2 by phosphorylation. In turn, LATS1 and LATS2 inhibit YAP/TAZ-dependent gene regulation through inhibitory phosphorylation of multiple serine residues, which prevents nuclear translocation of YAP/TAZ, and so interaction with TEAD1–TEAD4 (Hansen et al., 2015). However, YAP/TAZ and TEAD1–TEAD4 are not only regulated by the Hippo pathway, but also by many other signalling systems, including the AMPK pathway, mechanotransduction, G protein-coupled receptors and Wnt signalling, that communicate with the Hippo pathway (Hansen et al., 2015). This is why the term ‘Hippo signal transduction network’ arguably best describes the overall signalling system whose main molecular function is transcriptional regulation through the TEAD1–TEAD4 transcription factors.

The *Vgll1–Vgll4* genes in mammals are homologous to the *vestigial* gene in fly and also bind TEAD family transcription factors, so VGLLs can operate in a similar manner to YAP and TAZ to organise transcription. Vestigial and VGLL proteins use their TONDU domain (Vaudin et al., 1999) to bind to TEAD proteins via two interfaces at the same TEAD site also bound by YAP via three interfaces (Pobbati et al., 2012). Loss of *vestigial* reduces the wings of flies to vestiges (reviewed in Simon et al., 2016), which requires the TEAD homologue *scalloped* (Halder et al., 1998). In mammals, *Vgll1–Vgll3* have one TONDU domain, and are expressed in a tissue-specific fashion, while *Vgll4* has two TONDU domains and is ubiquitously expressed. Functionally, VGLL4 is a YAP-TEAD repressor or antagonist, whereas VGLL1–VGLL3 have been suggested to be ‘selector’ genes that specify cell and tissue types (Koontz et al., 2013). Recently, ETS1 was identified as an additional VGLL3-binding transcription factor, suggesting that TEAD proteins are not the only transcription factors that are co-regulated by VGLLs (Simon et al., 2017). As transcriptional regulation through TEAD1–TEAD4 is the output of Hippo-related signalling, then this suggests that VGLL1–VGLL4 integrate into the wider Hippo signal transduction network.

VGLLs have been linked to skeletal muscle. Fly *vestigial* is implicated in flight muscle development (Bernard et al., 2009). In mammals, *Vgll2* (also known as *Vito-1*) and *Vgll3* (also known as *Vito-2*) have been studied in relation to skeletal muscle, especially by the Braun and Stewart groups (Chen et al., 2004; Gunther et al., 2004; Maeda et al., 2002; Mielcarek et al., 2002, 2009)*. Vgll2* has an important role in adult muscle *in vivo*, as *Vgll2^−/−^* mice have a higher number of fast-twitch type IIb muscle fibres and downregulation of the *Myh7* slow type I gene (Honda et al., 2017). *Vgll3^−/−^* mice have a sex-linked immune phenotype (Liang et al., 2017) and a shortened QT interval (http://www.mousephenotype.org/data/genes/MGI:1920819) but it is unknown whether knockout of *Vgll3* affects myogenesis or adult skeletal muscle. Copy number alterations of *VGLL3* and *YAP1* have been reported for human soft tissue sarcomas including rhabdomoysarcomas, where VGLL3 is required for proliferation (Cancer Genome Atlas Research Network, 2017; Hélias-Rodzewicz et al., 2010).

Here, we analyse the regulation and molecular/cellular function of VGLL3 in skeletal muscle *in vitro* and *in vivo*, with reference to the roles of YAP and TAZ. We report that *Vgll3* was expressed at low levels in healthy muscle but that expression increased both during muscle hypertrophy and muscle regeneration. In disease settings, *VGLL3* was also highly expressed in dystrophic muscle and alveolar rhabdomyosarcoma. *Vgll3* expression increases as human and murine myoblasts undergo myogenic differentiation *ex vivo*. *VGLL3* knockdown suppressed myoblast proliferation, while constitutive *VGLL3* expression enhanced myogenic differentiation. However, adult skeletal muscle fibre types and structure were unaffected in *Vgll3*-null mice, presumably due to redundancy and/or feedback signalling. Proteomics revealed that VGLL3 binds the transcription factors TEAD1, TEAD3 and TEAD4 in myoblasts and/or myotubes, but no interactions were detected with major regulatory systems, such as kinases from various signalling pathways. Transcriptomic analysis shows that VGLL3 regulates the Hippo negative-feedback loop, and affects expression of genes controlling myogenesis including *Myf5*, *Pitx2/3*, genes encoding certain Wnts and IGF-binding proteins (IGFBPs). Thus we conclude that VGLL3 is a transcriptional co-factor that operates in parallel with the Hippo effectors YAP and TAZ to control myoblast proliferation and differentiation.

## RESULTS

### VGLL2 and VGLL3 expression in skeletal muscle and cancer

**Fig. 1.**
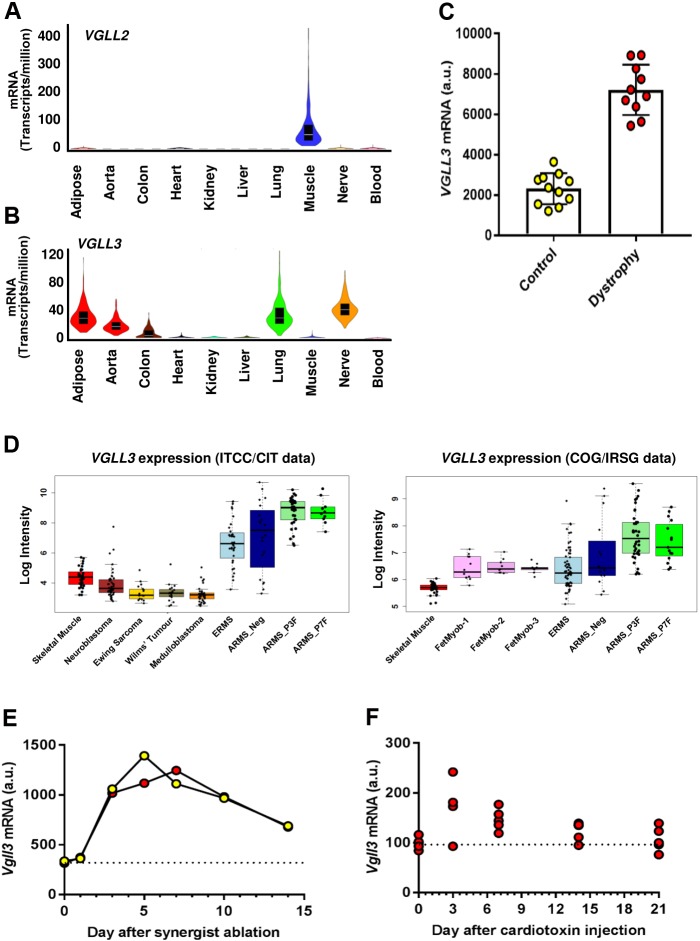
**VGLL2 and VGLL3 expression in skeletal muscle and cancer.** (A) *VGLL2* and (B) *VGLL3* expression in different human tissues according to an analysis of previously published data (G-TEx-Consortium, 2015). Violin plot with the box showing the 25–75th percentiles, with the median indicated. Outliers are excluded. (C) *VGLL3* expression in healthy and Duchenne muscular dystrophy quadriceps muscle biopsy samples according to data from Haslett et al. (2003). (D) VGLL3 expression in human skeletal muscle and various cancers including embryonal rhabdomyosarcoma (ERMS), fusion gene-negative alveolar rhabdomyosarcoma (AMRS_NEG), ARMS expressing PAX3-FOXO1 (ARMS_P3F) or PAX7-FOXO1 (ARMS_P7F) fusion genes according to data from Davicioni et al. (2009) and Williamson et al. (2010). In addition, VGLL3 expression in fetal myoblasts (FetMyob) at different days (1–3) is shown. Data is shown as a box plot, where the box represents the 25–75th percentiles, and the median is indicated. The whiskers show the 25th percentile minus 1.5×IQR and 75th percentile plus 1.5×IQR, with dots representing individual values. (E) Expression of *Vgll3* mRNA in mouse plantaris following synergist ablation according to data from Chaillou et al. (2013) or (F) regenerating mouse tibialis anterior muscle following cardiotoxin injection according to data from Lukjanenko et al. (2013), where the dotted line indicates levels in control muscle. a.u., arbitrary units.

The Hippo effector YAP, as well as VGLL1 and VGLL4, are able to bind TEAD family transcription factors (Koontz et al., 2013; Pobbati et al., 2012). However, although is it known that YAP is regulated by a plethora of signalling mechanisms, including phosphorylation, methylation, ubiquitylation, sumoylation and intracellular localization changes, far less is known about regulation of VGLLs. We therefore investigated regulation of VGLL3 and VGLL2, a paralogue that is highly expressed in skeletal muscle. While *VGLL2* is clearly expressed at higher levels in human muscle, VGLL3 is not (Fig. 1A,B). To examine whether VGLL3 and VGLL2 are regulated transcriptionally, we analysed published datasets from the GEO (see also Fig. S1) and supplementary datafiles from published papers. This revealed that *VGLL3* expression is >3-fold higher in the quadriceps of boys with Duchenne muscular dystrophy compared to healthy quadriceps (Fig. 1C; by contrast, *VGLL2* levels are similar between healthy and Duchenne individuals, see Supplementary Data S1A in Haslett et al., 2003). There is also robust *VGLL3* expression in human fetal myoblasts (Fig. 1D). In mice, the mean *Vgll3* expression increases both in synergist ablation-loaded hypertrophying plantaris muscle (Chaillou et al., 2013) and in cardiotoxin-induced regenerating tibialis anterior muscle (Lukjanenko et al., 2013) (Fig. 1E,F). In contrast, *Vgll2* declines in both situations (Fig. S1).

An earlier study reported that the *PAX3-FOXO1* fusion gene induced *Vgll3* expression by 8-fold in the forelimb buds of mouse embryos at embryonic day (E)10.5 (Lagha et al., 2010). Given that alveolar rhabdomyosarcomas (ARMS) frequently carry *PAX3-FOXO1* or *PAX7-FOXO1* fusion genes, we also analysed *VGLL3* expression in ARMS (Missiaglia et al., 2012; Shern et al., 2014). This showed that average *VGLL3* expression is highest in *PAX3-FOXO1* alveolar rhabdomyosarcomas when compared to other forms of rhabdomyosarcoma, fetal myoblasts or differentiated muscle (Fig. 1D). Finally, *Vgll3* expression is 2.3-fold higher in mouse embryonal rhabdomyosarcomas caused by YAP1 S127A expression in activated satellite cells than in control skeletal muscle (Tremblay et al., 2014).

In mouse muscle, a VGLL2 serine 261 phosphopeptide is detectable, but no VGLL3 phosphopeptides (Potts et al., 2017). This is relevant since fly Vestigial is phosphorylated at Ser215 by a p38 kinase (Pimmett et al., 2017). In human muscle, neither VGLL2 nor VGLL3 phosphopeptides are detectable pre- or post-intensive exercise (Hoffman et al., 2015). Together, this suggests that VGLL3 and VGLL2 are mainly regulated through their protein levels via transcription, rather than via post-translation modifications.

### VGLL3-binding protein partners in murine myoblasts and myotubes

**Fig. 2.**
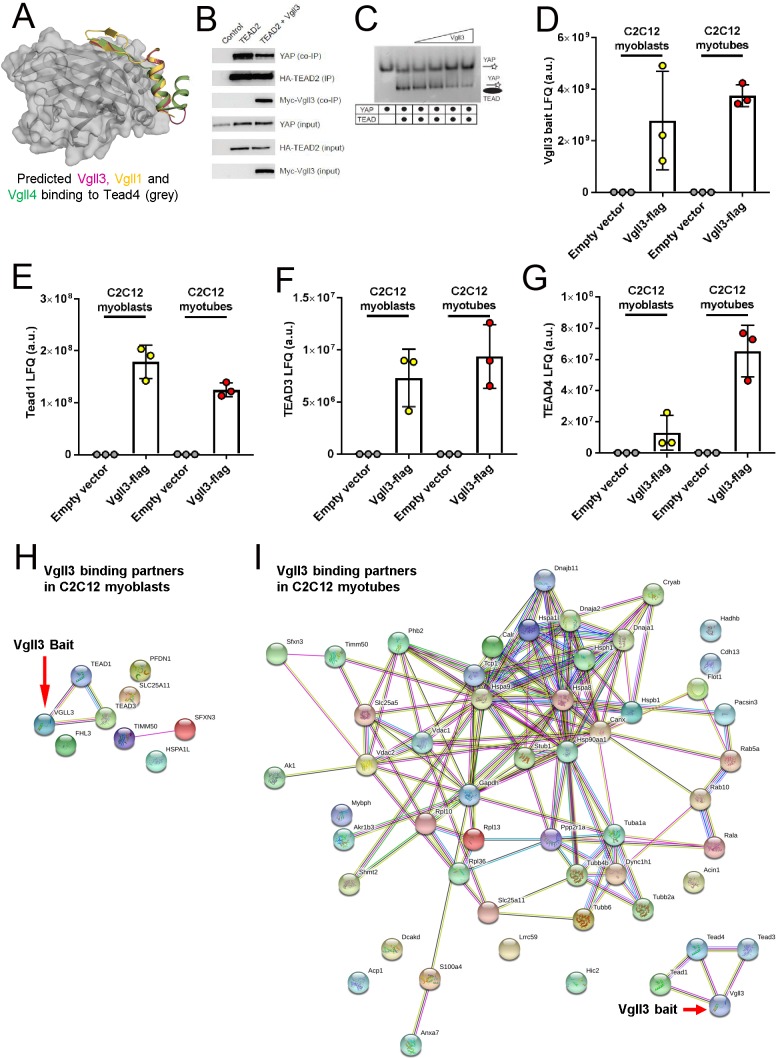
**VGLL3-binding protein partners in murine myoblasts and myotubes.** (A) Superimposition of VGLL1–TEAD4 (PDB ID: 5Z2Q) and VGLL4–TEAD4 (PDB ID: 4LN0) crystal structures with the modelled VGLL3–TEAD4 structure. A surface-ribbon representation of TEAD4 is shown in grey. (B) Expression of VGLL3 in HEK293 cells in an *in vitro* competition assay reduces the binding of YAP to TEAD2, suggesting competition between VGLL3 and YAP for binding to TEAD2. (C) Native gel showing the migration of free and TEAD4-bound labelled YAP (see diagram on right of gel). Only the labelled YAP is seen. Upon addition of VGLL3 peptide, the amount of TEAD4-bound YAP is reduced, with a concomitant increase in the amount of free YAP, again indicating competition between VGLL3 and YAP for binding to TEAD4. (D–G) VGLL3–Flag immunoprecipitation (IP) proteomics shows that the VGLL3 bait is detected (D) and that VGLL3–Flag binds TEAD1, TEAD3 and TEAD4 in murine C2C12 myoblasts and/or myotubes (E–G). (H) String analysis of VGLL3–Flag-binding partners identified in C2C12 myoblasts and (I) in C2C12 myotubes. a.u., arbitrary units; LFQ, label-free quantification.

In fly, Vestigial protein binds the transcription factor scalloped (the orthoparalogue of mammalian TEAD1– TEAD4) to create ‘wing cell-specific DNA-binding selectivity’ for Scalloped (Halder and Carroll, 2001; Halder et al., 1998). VGLL proteins also interact with the TEAD family of transcription factors; the structure of the core complex of VGLL1 and TEAD4 reveals that the TONDU domain in VGLL1 interacts with TEAD4 by forming two interfaces (Pobbati et al., 2012). Subsequently, VGLL4 has been also shown to pair with TEAD by forming similar interfaces (Jiao et al., 2014). The TONDU domains in *Drosophila* Vestigial and VGLL proteins are highly conserved. Thus, the core complex structure of VGLL3 with TEAD should be similar to that of VGLL1 or VGLL4. To test our prediction, we modelled the VGLL3–TEAD4 structure using the PHYRE2 protein fold recognition server. Superimposition of VGLL1–TEAD4 and VGLL4–TEAD4 crystal structures with the modelled VGLL3–TEAD4 structure shows that all three binary core complexes are structurally similar (Fig. 2A). To test experimentally whether VGLL3 and TEAD2 interact, we overexpressed HA–TEAD2 with Myc–VGLL3 and their known interactor YAP, in HEK293 cells and then immunoprecipitated HA–TEAD2, Myc–VGLL3 or YAP (Fig. 2B). This demonstrated that VGLL3 binds TEAD2, and that the presence of Myc–VGLL3 reduces binding of YAP to TEAD2, suggesting that Myc–VGLL3 and YAP compete to bind HA–TEAD2. By using a fluorescently labelled YAP and TEAD4, we also verified that VGLL3 directly competes with YAP (Fig. 2C). Addition of TEAD4 to labelled YAP results in the formation of a YAP–TEAD4 complex that could be monitored in a native gel. Introduction of TONDU domain-containing VGLL3 peptide reduces the amount of YAP–TEAD4 complex in a dose-dependent manner (Fig. 2C), suggesting a direct competition between YAP and VGLL3 for binding to TEAD4.

To systematically characterise VGLL3-binding proteins in myogenic cells in a non-biased fashion, we overexpressed and co-immunoprecipitated VGLL3–Flag from murine C2C12 myoblasts and myotubes and identified VGLL3-binding proteins through liquid chromatography and mass spectrometry (LC-MS). Identification of the bait VGLL3–Flag (Fig. 2D) shows that immunoprecipitation worked. In addition, identification of TEAD1 (Fig. 2E), TEAD3 (Fig. 2F) and TEAD4 (Fig. 2G) confirms that VGLL3 can bind TEAD family transcription factors in myoblasts and myotubes. Binding to TEAD isoforms was different depending on the state of myogenic differentiation. The VGLL3–TEAD1 and VGLL3–TEAD3 interactions did not change overtly between myoblasts and myotubes. Interaction between VGLL3 with TEAD4, however, was greater in myotubes, indicating that this complex likely has a role in the regulation of differentiation. Importantly, we did not identify other transcription factors among the co-immunoprecipitated proteins.

VGLL3 interaction proteomics also identified other proteins that interact with VGLL3 or through which VGLL3 additionally exerts its function. VGLL3 only bound to eight proteins in C2C12 myoblasts, but to 52 proteins in C2C12 myotubes. In addition to TEAD1, TEAD3 and TEAD4 (Fig. 2E–G), VGLL3 also binds these classes of proteins in myoblasts and/or myotubes (for a full protein list, see Table S1): (1) heat shock and related proteins (HSP90AA1, HSPA1L, HSPA8, HSPA9, HSPB1, HSPH1, CRYAB, DNAJA1, DNAJA2 and DNAJB11); (2) tubulins (TUBA1A, TUBB2A, TUBB4B and TUBB6); (3) metabolic enzymes/proteins (AK1, AKR1B1, GAPDH and HADHB); (4) mitochondrial channel proteins (VDAC1, VDAC2, SLC25A11, SLC25A5 and TIMM50); and (5) Ras-related proteins (RAB10, RAB5A and RALA).

It should be noted however, that heat shock proteins, tubulins and metabolic proteins, such as GAPDH, are frequently detected in proteomic studies (Wang et al., 2009) and may be proteins generally linked to the synthesis and folding of proteins.

We illustrate the functional interaction between VGLL3-binding proteins for C2C12 myoblasts (Fig. 2H) and C2C12 myotubes (Fig. 2I). The clear difference in the VGLL3 interactome in myoblast versus myotubes suggests that the role of VGLL3 in myogenic differentiation is at least partially regulated by changes in protein–protein interactions. Most prominent is the differential interaction with the TEAD isoforms, with VGLL3 interaction with TEAD1 and TEAD3 in myoblasts, but TEAD1, TEAD3 and, importantly, TEAD4 in myotubes. This is consistent with our previous observations showing that *Tead4* mRNA, and TEAD1 and TEAD4 protein increase markedly during myogenic differentiation (Sun et al., 2017). Changes in VGLL3-mediated transcriptional activation of target genes are thus likely related to this differential availability of TEAD isoforms. Overall, there was only minimal overlap with YAP and TAZ-binding proteins with the exception of TEAD1, TEAD3 and TEAD4 (Table S2 and Sun et al., 2017). Moreover, most putative binding partners of VGLL3 normally reside in the cytosol. Whereas many of these proteins are likely associated with the synthesis of VGLL3, our results might also mean that VGLL3 can localise to both the nucleus and cytosol. We used the nuclear export signal-prediction programme NetNes 1.1 to search for a nuclear export signal in the human VGLL3 amino acid sequence. This revealed a ‘MQDSLEVTL’ (single amino acid code) nuclear export signal that was fully conserved between man, chimpanzee, cat and mouse (Table S3).

### Transcriptomic analysis of VGLL3 target gene expression

**Fig. 3.**
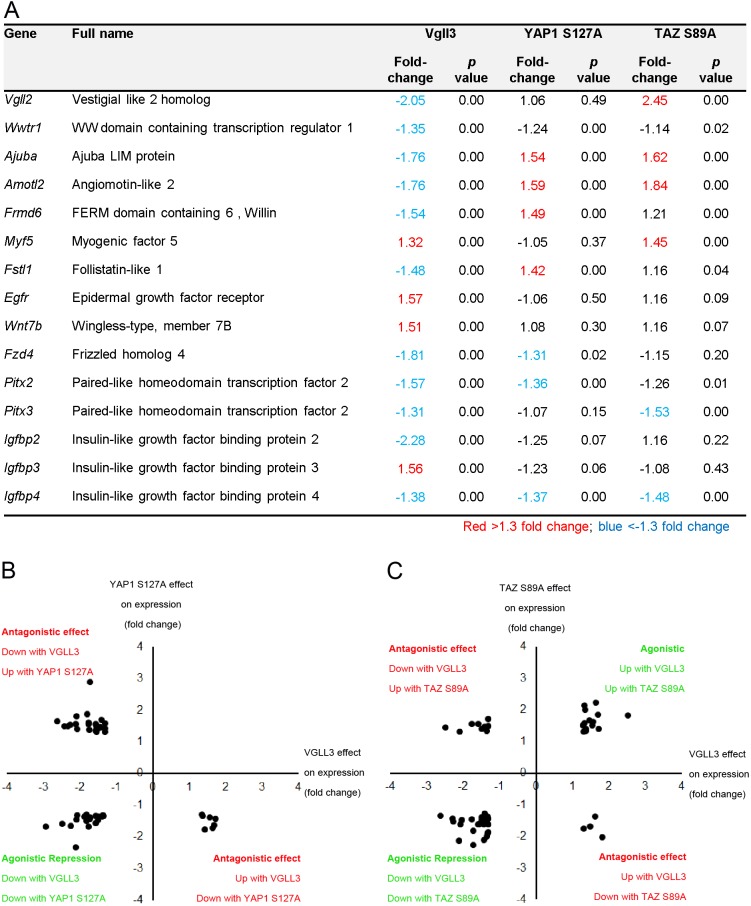
**Transcriptomic analysis of VGLL3 target gene expression.** (A) Examples of regulation of Hippo-related genes and of genes controlling muscle functions by VGLL3 versus YAP1 S127A or TAZ S89A in primary murine satellite cell-derived myoblasts at 48 h (FDR <10%). (B,C) In satellite cell-derived myoblasts, VGLL3 drives expression of genes that are both regulated and unregulated by YAP (B) or TAZ (C), having both agonist and antagonist effects.

Given that VGLL3 binds the same TEAD1, TEAD3 and TEAD4 transcription factors that are also bound by YAP and TAZ (Sun et al., 2017), we next assessed the effect of VGLL3 on gene expression in murine primary satellite cell-derived myoblasts. We compared the effects of VGLL3 on gene expression to our previous transcriptomic examination using YAP1 S127A or TAZ S89A (Sun et al., 2017). YAP1 S127A/TAZ S89A mutants are constitutively active because a serine residue is mutated to an alanine so that YAP or TAZ can no longer be inhibited by Ser127 or Ser89 phosphorylation, respectively, which normally prevents nuclear localisation. Using a cutoff difference of ≥30% or ≤30% and false discovery rate (FDR) <10%, we found that VGLL3 only induced the expression of one gene and downregulated that of nine genes in myoblasts after 24 h. However, VGLL3 enhanced the expression of 29 genes and lowered expression of 126 genes after 48 h (Fig. 3A; a full list of VGLL3-regulated genes is given in Table S4). Since VGLL3 represses 5.2 times as many genes as it induces, this identifies VGLL3 as a protein that mainly represses its target genes expression in myoblasts.

As predicted, many of the VGLL3-regulated genes are also regulated by YAP1 S127A and TAZ S89A (Sun et al., 2017; listed in Table S5). To illustrate this, we plotted all genes whose expression was significantly (FDR <10%) upregulated (by ≥30%) or downregulated (by ≤30%) in response to VGLL3 overexpression. Many of the genes downregulated by VGLL3 are upregulated by YAP S127A and vice versa, suggesting an antagonistic effect (Fig. 3B; Table S5). There are, however, genes that are downregulated by both YAP1 S127A and VGLL3 (Fig. 3B, lower left). Interestingly, VGLL3 or YAP1 S127A never induced the same gene (Fig. 3B). In contrast, VGLL3 and TAZ S89A could both induce the same genes, but again, there was no clear agonist–antagonist relationship (Fig. 3C; Table S5).

VGLL3 negatively regulates (≥1.3 fold and FDR <10%) many crucial genes of the Hippo signal transduction network including the Hippo transcriptional co-factors *Wwtr1* (encoding TAZ: down by 1.4-fold) and *Vgll2* (down by 2.1-fold), in addition to also suppressing *Ajuba*, *Amotl2* and *Frmd6* (down 1.5–1.8 fold) (Fig. 3A; Tables S4 and S5). As TAZ S89A drives expression of *Vgll2* and *Vgll3* (Sun et al., 2017), this suggests that VGLL3, TAZ and VGLL2 cross-regulate each other. Other downregulated genes include *Fzd4F*, the myokine *Flst1*, the paired-like homeodomain transcription factors *Pitx2* and *Pitx3*, and the IGFBPs *Igfbp2* and *Igfbp4.* By contrast, VGLL3 promotes expression of the myogenic regulatory factor *Myf5*, the growth factor receptor *Egfr*, the Wnt pathway member *Wnt7b* and the IGFBP *Igfbp3* (Fig. 3A and Tables S4 and S5).

Genes identified as downregulated by VGLL3 overexpression from our transcriptomic studies in mouse (Fig. 3) were also analysed in human myoblasts and myocytes using quantitative real-time RT-PCR (qRT-PCR) after VGLL3 knockdown or overexpression (Table S6). Most genes deregulated by VGLL3 overexpression in mouse were validated as also being affected in this human system, with downregulation of *VGLL2*, *AJUBA*, *AMOTL2*, *FSTL1* and *FZD4*, with *EGFR1* also repressed, while *WWTR1* was induced, and *FRMD6* and *PITX2/3* were unchanged (Table S6).

### VGLL3 expression dynamics and localisation during myogenesis

**Fig. 4.**
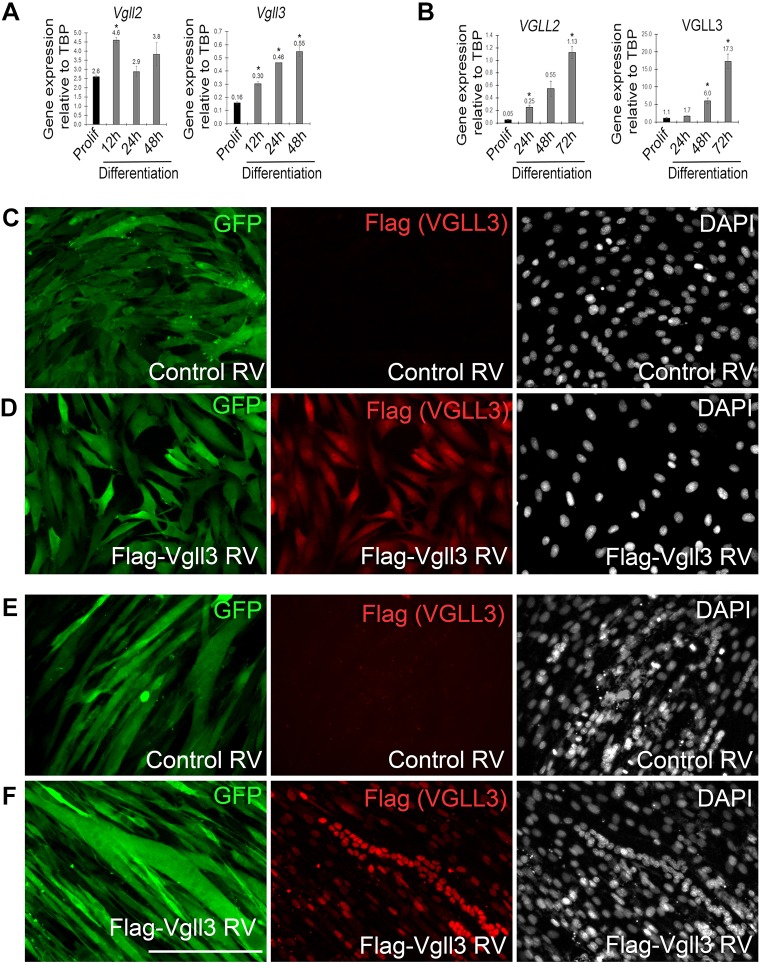
**VGLL3 expression dynamics and localisation during myogenesis.** (A) Expression of *Vgll2* and *Vgll3* as analysed by qRT-PCR in proliferating and differentiating primary mouse satellite cell-derived myoblasts or (B) primary human myoblasts *ex vivo*. Data are presented as mean±s.e.m., where *n*=3 mice or independent experiments. *P<0.05 between proliferation and differentiation samples (paired two-tailed *t*-test). (C,D) Proliferating murine C2C12 myoblasts transduced with (C) control or (D) Flag–VGLL3-encoding retrovirus, and immunolabelled for eGFP to identify transduced cells (from the *IRES-eGFP* in the retroviral backbone) and Flag to detect Flag–VGLL3. (E,F) Differentiated C2C12 myocytes and multinucleated myotubes transduced with (E) control or (F) Flag–VGLL3-encoding retrovirus (RV), and immunolabelled for eGFP and Flag to detect Flag–VGLL3, showing clear nuclear localisation of VGLL3. Scale bar: 100 µm.

To study expression of *Vgll2* and *Vgll3* in mouse satellite cells and human primary myoblasts during myogenic progression, we first performed qRT-PCR. Generally *Vgll2* and *Vgll3* expression increased during myogenic differentiation, with *Vgll3* increased in both mouse and human differentiating myoblasts, while *Vgll2* only increased transiently in mouse (Fig. 4A,B). In humans, *VGLL2* is selectively expressed in skeletal muscle, whereas *VGLL3* expression is low in muscle compared to other tissues (Fig. 1A,B). To analyse protein distribution, we purchased commercially available anti-VGLL3 antibodies, but found immunolabelling unchanged upon retroviral-mediated constitutive VGLL3 expression (data not shown). Instead, we designed a retroviral vector encoding *Flag–Vgll3* and used anti-Flag antibodies to detect the Flag-tagged VGLL3 protein. This showed that VGLL3 was located in both the nucleus and cytoplasm of C2C12 murine myoblasts (Fig. 4C,D). Upon myogenic differentiation into myocytes and multi-nucleated myotubes, however, there was a clear and robust nuclear localisation of Flag-tagged VGLL3 (Fig. 4E,F).

### VGLL3 supports proliferation and differentiation in mouse and human myoblasts

**Fig. 5.**
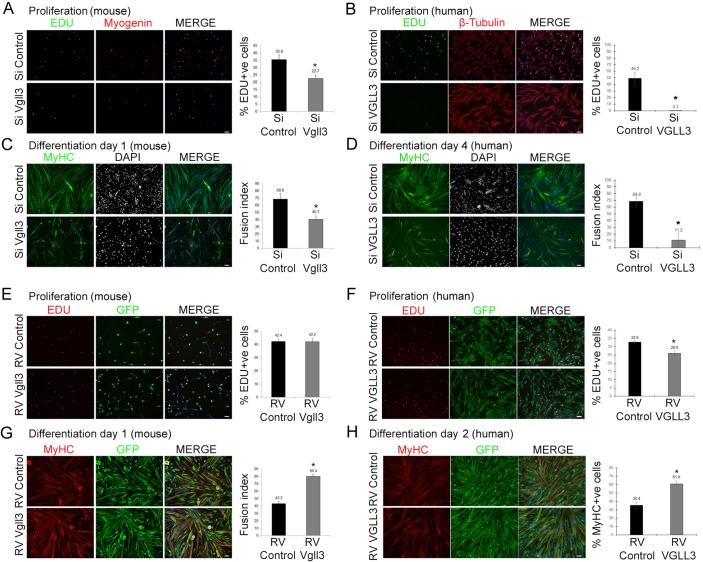
***Vgll3* supports proliferation and differentiation in mouse and human myoblasts.** (A–D) Effects of siRNA-mediated *Vgll3* knockdown on cell proliferation (A,B) and myotube formation (C,D) in primary murine satellite cell-derived myoblasts (A,C) or immortalised human C25Cl48 myoblasts (B,D). Proliferating mouse or human myoblasts were immunolabelled for (A) myogenin or (B) β-tubulin, and EdU incorporation was measured. (C,D) Myotubes were visualized by MyHC immunolabelling. (E–H) Effects of retroviral-mediated VGLL3 overexpression on myoblast proliferation (E,F) and myotube formation (G,H) in both transiently transduced primary murine satellite cell-derived myoblasts (E,G) or stable *VGLL3*-expressing immortalised human C25Cl48 myoblasts (F,H). (E,F) Proliferating myoblasts were immunolabelled for GFP (to detect transduced cells from the *IRES-eGFP* in the retroviral backbone) and EdU incorporation measured, while (G,H) myotubes were immunolabelled for GFP and MyHC. Reduction of VGLL3 generally inhibits proliferation, while constitutive VGLL3 expression enhances myogenic differentiation. Data are presented as mean±s.e.m., where *n*=3 mice or independent experiments. *P<0.05 between either si Vgll3 compared to si Control (A–D), or RV VGLL3 compared to RV Control (E–H) (two-tailed *t*-test). Scale bars: 100 µm.

To assess VGLL3 function in myoblasts, we first employed siRNA-mediated downregulation of VGLL3, which significantly reduced proliferation of both primary murine satellite cell-derived myoblasts and the human C25Cl48 immortalised myoblast line (Mamchaoui et al., 2011) (Fig. 5A,B). This was particularly striking in human myoblasts, where 5-ethynyl-2′-deoxyuridine (EdU) incorporation after a 2 h EdU pulse dropped from 49.2% to 0.3% when VGLL3 was knocked down (Fig. 5B), suggesting that VGLL3 is essential for proliferation of human myoblasts. We next evaluated the impact of VGLL3 knockdown on myogenic differentiation into multinucleated myotubes and the assembly of sarcomeres. VGLL3 downregulation reduced murine myotube formation, as indicated by a drop in the proportion of nuclei within myotubes (fusion index) from 68.8% to 40.7% (Fig. 5C) and in human myoblasts from 68.8% to 11.2% (Fig. 5D). However, especially in human myoblasts, the reduced differentiation measured in VGLL3 downregulated cells may be largely indirect, being at least partially explained by the presence of fewer myoblasts owing to their reduced proliferation rate, even though siRNA-transfected cells were replated at the same density as controls before inducing differentiation.

We then examined retroviral-mediated overexpression of VGLL3 on proliferation and differentiation in transiently transduced primary murine satellite cell-derived myoblasts and stable *VGLL3*-expressing human C25Cl48 immortalised myoblasts. VGLL3 overexpression did not further increase mouse satellite cell proliferation (Fig. 5E), but in human myoblasts, the proportion of EdU-positive myoblasts overexpressing VGLL3 dropped slightly to 26.0%, from 32.8% in myoblasts transduced with control retrovirus (Fig. 5F). VGLL3 overexpression increased the fusion index in mouse satellite cell-derived myoblasts to 80.4%, from 43.2% with the control (Fig. 5G) and to 61.0% from 35.4% in human myoblasts (Fig. 5H). Together, these results in mouse and human myoblasts show that *Vgll3* expression enhances myoblast differentiation.

### VGLL3-null mice have normal skeletal muscle and myoblast function

**Fig. 6.**
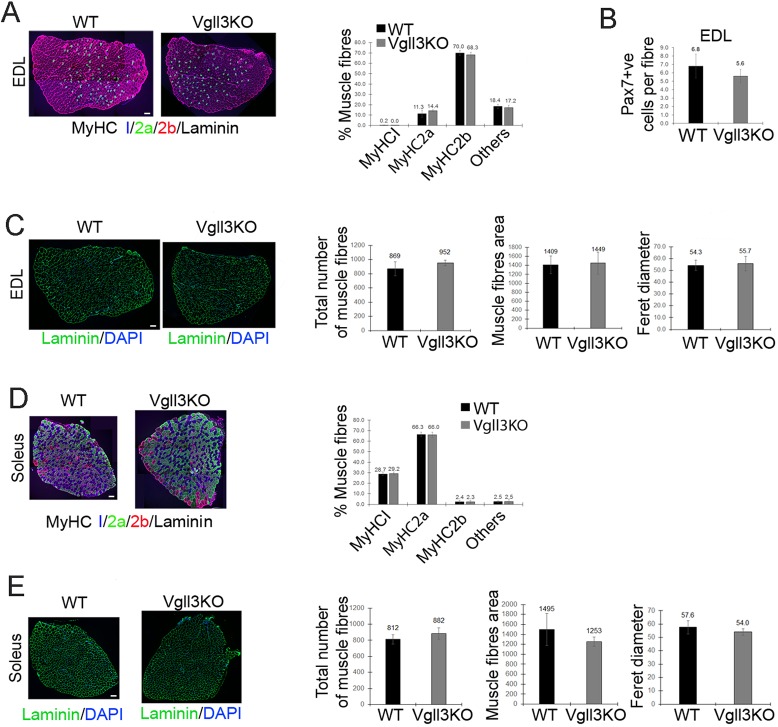
***Vgll3*-null mice have normal muscle structure and fibre type content.** (A) Extensor digitorum longus (EDL) or (D) Soleus muscle transverse sections co-immunolabelled for myosin heavy chain (MyHC) type I, MyHC type 2a or MyHC type 2b, together with laminin, from wild-type (WT) or *Vgll3*^−/−^ (Vgll3KO) mice. Quantification from such images reveals no changes in the proportions of each muscle fibre type. (B) Quantification of the number of Pax7-expressing satellite cells per isolated EDL myofibre in wild-type and *Vgll3*^−/−^ mice. (C) EDL or (E) Soleus muscle transverse sections immunolabelled for laminin and counterstained with DAPI from wild-type or *Vgll3*^−/−^ mice and corresponding quantification of the total number of myofibres per muscle, together with area and Feret diameter of muscle fibres. Data are presented as mean±s.e.m. from *n*=3 mice. *P<0.05 between Vgll3KO and WT (unpaired two-tailed *t*-test). Scale bars: 50 µm.

**Fig. 7.**
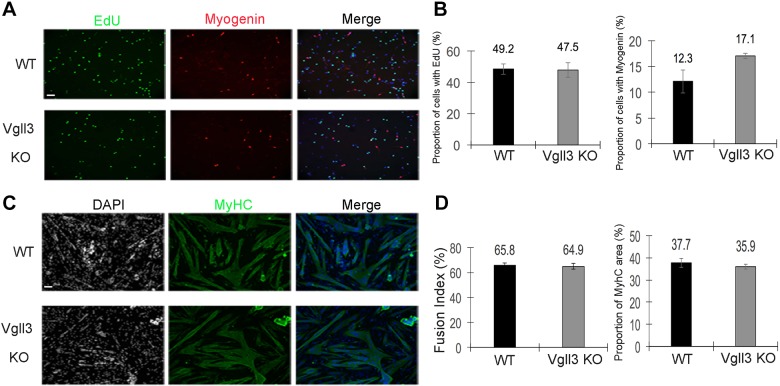
**Loss of VGLL3 does not affect proliferation or differentiation of satellite cell-derived myoblasts.** (A) Primary satellite cells were isolated from the EDL muscle of wild-type (WT) or *Vgll3^−/−^* (Vgll3 KO) mice. Myoblasts were expanded *in vitro* (on Matrigel) in proliferation medium and pulsed with EdU for 2 h. EdU incorporation was visualised and immunolabelling for myogenin performed. (B) Proliferation of *Vgll3^−/−^* satellite cells was similar to that of wild-type (WT) controls, with 48–49% of myoblasts incorporating EdU. There was a non-significant trend for more myogenin-positive cells in the *Vgll3^−/−^* satellite cells compared to control. (C) EDL Primary satellite cells of WT or *Vgll3^−/−^* mice were expanded *in vitro* (on Matrigel) in proliferation medium and then switched to differentiation medium for 1 day. Cells were immunolabelled for myosin heavy chain (MyHC), then the fusion index and MyHC-positive area determined. (D) Myotube formation in *Vgll3^−/−^* satellite cells was not significantly different to wild-type control satellite cells, with a fusion index of 64.9% compared to 65.8% in controls. Data are presented as mean±s.e.m. for *n*=3 mice. *P<0.05 between Vgll3 KO compared to WT (unpaired two-tailed *t*-test). Scale bars: 50 µm.

To examine the role of *Vgll3* in skeletal muscle development and maintenance *in vivo*, we characterised skeletal muscle of hind limbs of *Vgll3^−/−^* mice, generated as part of the International Phenotyping Consortium (Brown and Moore, 2012). *Vgll3^−/−^* mice were viable, born in the expected Mendelian ratios and did not show any overt phenotype while reaching adulthood. The extensor digitorum longus (EDL) and soleus muscles of adult *Vgll3^−/−^* mice and wild-type controls were analysed independently by the London (Fig. 6) and Munich (Fig. S2 and Table S7) teams. We found no significant difference between the EDL and Soleus of *Vgll3^−/−^* and wild-type control mice in fibre type distribution (Fig. 6A,D), the number of satellite cells per muscle fibre (Fig. 6B), and fibre numbers and size (Fig. 6C,E). This shows that the constitutive loss of VGLL3 function can be compensated for *in vivo*, suggesting that VGLL3 is not essential for normal skeletal muscle development. Interestingly, satellite cell-derived myoblasts isolated from *Vgll3^−/−^* mice did not show any changes in proliferation rate compared to wild-type controls, as determined by measuring EdU incorporation following a 2 h pulse (Fig. 7A,B). Expression of *Pax7*, myogenin (*Myog*), *Vgll2* and *Vgll4* were also unchanged in *Vgll3^−/−^* mice compared to wild-type controls (Fig. S3). Finally, differentiation of *Vgll3^−/−^* satellite cell-derived myoblasts was assessed by calculating the fusion index to test their ability to form multinucleated myotubes and measure the area occupied by MyHC-expressing myotubes, but again no statistically significant differences were found compared to wild-type controls (Fig. 7C,D).

## DISCUSSION

Here, we report a comprehensive analysis of the regulation, protein binding and effect on target gene expression of the transcriptional co-regulator VGLL3 in skeletal myogenesis, and compare VGLL3 with YAP and TAZ. Additionally, we characterise VGLL3 function in skeletal muscle *in vitro* and *in vivo*.

Whilst *Vgll2* is highly expressed in skeletal muscle and involved in myogenic differentiation (Chen et al., 2004) and muscle fibre type distribution (Honda et al., 2017), far less is known about regulation and function of VGLL3 in adult skeletal muscle. Generally, *Vgll3* is expressed at low levels in healthy muscle, but levels increase during regeneration (Lukjanenko et al., 2013) and in mechanically loaded, hypertrophying plantaris muscle (Chaillou et al., 2013). *VGLL3* is also differentially expressed human patients with muscle-related disease, with a >3-fold increase in quadriceps muscles of Duchenne muscular dystrophy patients (Haslett et al., 2003). Copy number gains of the *VGLL3* gene have also been reported for some rhabdomyosarcomas (Hélias-Rodzewicz et al., 2010) and in sarcomas (Cancer Genome Atlas Research Network, 2017; Hélias-Rodzewicz et al., 2010). Moreover, *VGLL3* expression is high in ARMS associated with *PAX3-FOXO1* and *PAX7-FOXO1* fusion genes (Missiaglia et al., 2012; Shern et al., 2014) when compared to other cancers and controls. High expression of VGLL3 in *PAX3-FOXO1* fusion gene-positive ARMS is intriguing because expression of the *PAX3-FOXO1* fusion gene in mouse embryos induces *Vgll3* expression in the forelimb bud at E10.5 by 8-fold (Lagha et al., 2010; Missiaglia et al., 2012; Shern et al., 2014). Furthermore, a ChIP-Seq analysis of *PAX3-FOXO1* target genes reported *VGLL3* as a direct *PAX3-FOXO1* target (Cao et al., 2010). Collectively, this suggests that VGLL3 is controlled at the transcriptional level when myoblasts proliferate or muscle hypertrophies. This contrasts with what is found for the Hippo effectors YAP and TAZ, which are regulated by an extensive Hippo signal transduction network that regulates via protein modification (phosphorylation; see Introduction).

In fly, *vestigial* binds the TEAD homologue *scalloped* (Halder and Carroll, 2001; Halder et al., 1998). VGLLs and TEADs also interact in mammals (Koontz et al., 2013; Pobbati et al., 2012) but recently ETS1 has been identified as a non-TEAD transcription factor that can be bound by VGLL3 (Simon et al., 2017). Our proteomic analysis of VGLL3–Flag-binding proteins in C2C12 myoblasts and myotubes revealed that VGLL3 competes for the same TEAD1, TEAD3 and TEAD4 transcription factors as bound by YAP and TAZ in these cells (Sun et al., 2017). The clear difference in the VGLL3 interactome in myoblast versus myotubes, however, indicates that the role of this protein in myogenic differentiation is, in part, regulated by changes in protein–protein interaction. This includes differential interaction with the TEAD isoforms, with interaction with TEAD1 and TEAD3 in myoblasts, but TEAD1, TEAD3 and TEAD4 in myotubes. This is consistent with previous observations on the expression dynamics of *Tead1–Tead4* mRNA, and TEAD1 and TEAD4 protein, where TEAD4 levels increase markedly during myogenic differentiation (Benhaddou et al., 2012; Sun et al., 2017). Indeed, TEAD4 knockdown inhibits myogenic differentiation in satellite cell-derived myoblasts (Sun et al., 2017). Changes in VGLL3-mediated activation of target genes between myoblasts and myotubes are thus likely related to this differential availability of the TEAD isoforms for activation by VGLL3.

TEAD1, TEAD3 and TEAD4 are the only proteins that are co-immunoprecipitated together with VGLL3, YAP and TAZ. Whereas YAP and TAZ share 18.6% of their binding partners, VGLL3 only shares ∼1% of the binding partners with those for YAP or TAZ. Moreover, we could only identify the phosphatase PPP2R1A as a potential protein-modifying regulator among the VGLL3-binding proteins. Interestingly, PPP2R1A is functionally relevant in ARMS (Akaike et al., 2018), where VGLL3 is highly expressed. With VGLL3, we additionally co-immunoprecipitated many cytosolic proteins, including proteins involved with protein processing in the endoplasmic reticulum, especially heat-shock proteins and components of the tubulin cytoskeleton. VGLL3, as assessed by using a Flag-tagged version, was present in both the nucleus and cytoplasm in myoblasts, so VGLL3 interaction with cytoplasmic proteins is likely. We also discovered the presence of a nuclear export signal in VGLL3. However, heat shock proteins, tubulins and metabolic proteins, such as GAPDH, are frequently detected in proteomic studies (Wang et al., 2009) and may be generally linked to the synthesis and folding of proteins such as Flag–VGLL3, although others might be false positives, such as Flag-binding proteins.

Given that VGLL3, YAP and TAZ compete for the same TEAD1, TEAD3 and TEAD4 transcription factors (Sun et al., 2017), do VGLL3, YAP and TAZ regulate the same genes as agonists or antagonists? We compared our new VGLL3 transcriptomic analysis in myoblasts to our earlier data, where we measured the effect of YAP1 S127A and TAZ S89A on gene expression (Sun et al., 2017). This revealed that ∼5 times more genes were downregulated by VGLL3 than induced, suggesting that VGLL3 acts mainly to reduce its target gene expression. In the VGLL3 interactome data, however, we found no obvious binding partner with a known repressor function. In contrast to VGLL3, YAP induces and represses similar numbers of genes (Judson et al., 2012; Sun et al., 2017). While there was a broad overlap between genes regulated by VGLL3, YAP1 S127A and TAZ S89A, there was no simple agonist or antagonist relationship. For example, VGLL3, YAP1 S127A and TAZ S89A all repress genes such as *Igfbp4*. In contrast, the Hippo gene *Amotl2* is downregulated by VGLL3 and induced by TAZ S89A and YAP1 S127A, suggesting that VGLL3 and YAP/TAZ act as antagonists in relation to *Amotl2* expression. Most genes deregulated by VGLL3 overexpression in mouse were also similarly affected by VGLL3 overexpression in human myoblasts.

When analysing individual genes, some gene groups and individual genes stood out. First, VGLL3 mainly downregulated the YAP-induced Hippo genes *Ajuba*, *Amotl2* and *Frmd6*, which are induced by YAP1 S127A, and which we have previously termed as encoding proteins that together form the Hippo negative-feedback loop (Judson et al., 2012). Additionally, VGLL3 repressed *Vgll2* in man and mouse, and also repressed the Hippo transcriptional co-factors *Wwtr1* (encoding TAZ) in mouse, but enhanced *WWTR1* in man. Differences in effects of VGLL3 on gene expression between mouse and man can possibly be explained by complexity of the TEAD transcriptional system. There are four TEAD transcription factors, YAP, TAZ and four VGLLs. These factors play a crucial role during myogenic progression (Sun et al., 2017; Watt et al., 2010) and change expression during differentiation (Sun et al., 2017 and this study). Such complex regulation also involves negative-feedback loops regulated by YAP/TAZ and TEAD1–TEAD4 (Judson et al., 2012). Thus, depending on protein levels, the state of differentiation, and activity of the negative-feedback loop, expression of target genes can vary between species.

In addition to the Hippo-related genes, VGLL3 and TAZ S89A also both induce the myogenic regulatory factor *Myf5*. This may be important for embryonic muscle development as the somite muscle precursor cell marker Pax3 induces *Vgll3* (Lagha et al., 2010). Pax3-positive cells then become myoblasts by expressing *Myf5*. Intriguingly, a key enhancer of *Myf5* has a TEAD-bound CATTCC/GGAATG element (Ribas et al., 2011) suggesting a Pax3–VGLL3–TEAD–Myf5 signalling axis that allows Pax3-positive precursors to turn into *Myf5*-expressing myoblasts could be active in the developing embryo. Future studies should explore this idea during *in vivo* embryogenesis.

Other VGLL3-regulated genes in mouse include the myokine *Flst1* (Ouchi et al., 2008) and *Egfr*, a growth factor receptor associated especially with embryonal rhabdomyosarcoma (Ganti et al., 2006). Other targets are the Wnt pathway members *Wnt7b* and *Fzd4*, which is relevant given that YAP and TAZ also regulate Wnt signalling genes or are regulated by Wnt members (Park et al., 2015; Sun et al., 2017). The paired-like homeodomain transcription factors *Pitx2* and *Pitx3* which are critical for fetal and regenerative myogenesis (Knopp et al., 2013; L'Honore et al., 2014) were also expressed at lower levels in myoblasts overexpressing VGLL3, as were the IGFBPs *IGFBP2* and *IGFBP4*, which fine-tune IGF-1 signalling (Allard and Duan, 2018). In summary, VGLL3 acts mainly to repress gene expression, for example acting on the genes comprising the Hippo negative-feedback loop, and regulating genes such as *Myf5* that control the muscle lineage.

After analysing the molecular regulation and function of VGLL3, we tested the effect of VGLL3 gain- and loss-of-function on mouse and human myoblasts, as well as the skeletal muscle phenotype of *Vgll3*^−/−^ mice. Here, the most striking result is that the knockdown of *VGLL3* in human myoblasts almost completely blocks proliferation. A link between VGLL3 and proliferation at least in some cell types may explain the high expression, copy number gains and function of VGLL3 in sarcomas, including rhabdomyosarcomas (Cancer Genome Atlas Research Network, 2017; Hélias-Rodzewicz et al., 2010). Suppression of the Hippo negative-feedback loop by VGLL3 also places VGLL3 as a promoter of proliferation. Conversely, *VGLL3* has been reported to act as a tumour suppressor in epithelial ovarian cancer (Gambaro et al., 2013). This might reflect that VGLL3 can have both proliferation-promoting oncoprotein or pro-differentiation tumour suppressor effects. Indeed, we found that overexpression of *VGLL3* at a later time point promotes the differentiation of both mouse and human myoblasts. This is reminiscent of what is seen for TAZ (Mohamed et al., 2016; Sun et al., 2017), which can also switch from a pro-proliferation to a pro-differentiation regulator.

Given the molecular regulation and function of VGLL3 and its role in myoblast proliferation and differentiation, it is surprising that *Vgll3^−/−^* mice develop apparently normally without a detectable skeletal muscle phenotype. However, a sex-linked autoimmunity phenotype has been reported recently for *Vgll3^−/−^* mice (Liang et al., 2017). Reasons for the lack of a major phenotype *in vivo* could be the redundancy among TEAD family-targeting transcriptional co-regulators and the potent Hippo negative-feedback loop, which also operates in skeletal muscle cells (Judson et al., 2012). This feedback loop also probably prevents a major phenotype when the mouse *Yap1* gene is mutated in the genome so that the serine 112 phosphorylation site is mutated into an alanine (Chen et al., 2015). Similarly, we found no overt muscle phenotype in *Wwtr1^−/−^* (TAZ-null) mice (Sun et al., 2017). Redundancy between YAP, TAZ and VGLL1–VGLL4 may be important to prevent the mutation of a single gene causing the breakdown of an organ system. Here, a good example are the myogenic regulatory factors *MyoD1* and *Myf5* whose individual knockout has little effect on skeletal muscle development, but whose combined knockout essentially halts functional myogenesis (Kassar-Duchossoy et al., 2004; Rudnicki et al., 1993).

## MATERIALS AND METHODS

### Ethics statement for animal experimentation

Mice were bred in accordance with British law under the provisions of the Animals (Scientific Procedures) Act 1986, as approved by the King's College London Ethical Review Process committee. Wild-type mice were C57BL/10. *Vgll3*-knockout mice (VGLL3-DEL890INS2-EM1-B6N) were provided by the MRC (The Mary Lyon Centre, MRC Harwell Institute, UK). *Vgll3-*knockout mice had been engineered using the CRISPR/Cas9 technology to induce a deletion of 890 nucleotides from the *Vgll3* gene (including the functionally critical exon 2) to induce a premature stop codon and a null allele (C57BL/6NtTac genetic background).

### Analysis of published datasets

We downloaded several gene expression datasets from Gene Expression Omnibus (GEO) to evaluate whether challenges or disease affect *Vgll3* expression. These include dataset GDS4932 for gene expression in synergist ablation-overloaded, hypertrophying mouse plantaris muscle (Chaillou et al., 2013), dataset GDS4924 for regeneration after cardiotoxin-induced muscle injury of mouse tibialis anterior muscle (Lukjanenko et al., 2013) and dataset GDS612 from quadriceps skeletal muscle biopsies from DMD patients and unaffected control individuals (Haslett et al., 2003). Moreover, we used supplementary data files from studies comparing resting, resistance exercise or endurance exercise-trained human muscle (Vissing and Schjerling, 2014), investigating the effect of high-intensity exercise on the human muscle phosphoproteome (Hoffman et al., 2015) and investigating the effect of maximal contractions on the mouse muscle phosphoproteome (Potts et al., 2017). To examine *VGLL3* expression in cancer, we analysed gene expression in 235 rhabdomyosarcoma (RMS) patients from two publicly available datasets: containing 101 samples [Innovative Therapies for Children with Cancer/Carte d'Identite des Tumeurs (ITTC/CIT)] (Williamson et al., 2010), and 134 RMS samples [Children's Oncology Group/Intergroup Rhabdomyosarcoma Study Group (COG/IRSG)] (Davicioni et al., 2009).

### Modelling VGLLs binding to TEAD4 transcription factor

The structure of VGLL3 in the VGLL3–TEAD4 complex was predicted using the Phyre2 protein fold recognition server (Kelley et al., 2015). Phyre2 predicts the three-dimensional structure of the protein using its primary sequence. A model of the TONDU motif of VGLL3 was built using homology detection methods. The homologous crystal structure of the VGLL1–TEAD4 complex (PDB ID: 5Z2Q) was used in the prediction, and the confidence score of the predicted VGLL3 model is 99.8%. In Fig. 2A, the predicted VGLL3 model was overlaid on crystal structures of the VGLL1–TEAD4 complex (PDB ID: 5Z2Q) and VGLL4–TEAD complex (PDB ID: 4LN0). Only one TEAD is shown for clarity.

### *In vitro* cell culture

Immortalized human myoblasts (C25Cl48) were provided by Dr Vincent Mouly, Institute Myology, Paris, France (Mamchaoui et al., 2011). Proliferating human myoblasts were maintained in skeletal muscle cell growth medium (Promo-Cell, C-23160) supplemented with 20% FBS (Gibco). To induce differentiation, myoblasts were switched to the skeletal muscle cell differentiation medium (Promcell: C-23161). Proliferation and differentiation medium were supplemented with 50 µg/ml Gentamycin.

Mouse C2C12 myoblasts and HEK293T cells were cultured in DMEM (Sigma), supplemented with 10% FBS (Gibco). To induce differentiation, C2C12 myoblasts were cultured in growth medium until confluence, then the medium was switched to DMEM with 2% horse serum (Gibco).

### Competitive binding assay

HEK293 cells were grown in RPMI supplemented with 10% FBS at 37°C in 5% CO_2_. HEK293 cells were transfected with HA–TEAD2 and/or Myc–VGLL3, as indicated. The tags were introduced to the 5′ end through modification of the pCI-neo vector (Promega). Constructs were transfected using Lipofectamine 2000. At 24 h post transfection, the cells were lysed in buffer with 20 mM Tris-HCl pH 8.0, 150 mM NaCl and 0.1% Triton X-100 and protease inhibitors. HA–TEAD2 was immunoprecipitated using anti-HA beads (Sigma) and the co-immunoprecipitated YAP was detected using anti-YAP antibody (D8H1X, Cell Signaling). HA–TEAD2 was detected using anti-HA-HRP (Sigma) and Myc–VGLL3 was detected using clone 4A6 antibody (Millipore). For full details of antibodies, see Table S8.

Labelled human YAP (amino acids 61–100) and unlabelled human VGLL3 (amino acids 86–112) peptides were used. Histidine-tagged human TEAD4 (amino acids 217–434) was expressed in *E.coli* and purified by using immobilized metal affinity chromatography (IMAC) and gel filtration chromatography. TEAD4 was incubated with an increasing concentration of VGLL3 peptide prior to the addition of labelled YAP. After a 10-min incubation at room temperature, a native gel (10%) was run at 100 V to visualize free and TEAD-bound YAP.

### Proteomics

The preparation of samples and analysis was performed as described previously (Sun et al., 2017). Briefly, C2C12 cells were grown in Dulbecco's modified Eagle's medium (DMEM) with 10% fetal bovine serum (FBS) and 4 mM glutamine. For immunoprecipitation, 80,000 C2C12 cells were seeded per 10 cm dish. The following day, the cells were transduced with control or *Vgll3*-encoding retroviral supernatant. The next day, transduction was confirmed by assessing the presence of green fluorescent protein (GFP) from an *IRES-eGFP* in the retroviral backbone. Successful immunoprecipitation of Flag-VGLL3 was also confirmed by immunoprecipitation followed by western blotting using anti-Flag antibody (Sigma, F7425) (data not shown). Cells were split either into proliferation or differentiation conditions (72 h). Proliferating or differentiated cells were then washed on ice with PBS, and collected in lysis buffer (150 mM NaCl, 20 mM Tris-HCl pH 7.5, 1% Triton X-100) with 1 mM sodium orthovanadate, protease inhibitor cocktail (Sigma, p8340), and phenylmethylsulfonyl fluoride (PMSF) (Sigma). Lysates were incubated for 1 h on ice and centrifuged at 11,000 ***g*** at 4°C for 5 min and supernatant incubated at 4°C with anti-Flag M2 magnetic beads (Sigma, M8823). Beads were washed three times with washing buffer (150 mM NaCl, 20 mM Tris-HCl pH 7.5). Sample preparation/mass spectrometry were performed as described previously (Turriziani et al., 2014). The mass spectrometry proteomics data have been deposited to the ProteomeXchange Consortium via the PRIDE (Perez-Riverol et al., 2015; Vizcaíno et al., 2016) partner repository with the dataset identifier PXD012040.

### Gene expression

Primary murine myoblasts (isolated as described in Sun et al., 2017) were transduced with VGLL3-encoding retrovirus for 24 h or 48 h, with empty vector retrovirus as control. RNA was isolated using TRIzol (ThermoFisher Scientific) followed by purification and DNase digestion using RNeasy minikits (Qiagen, Venlo, Netherlands). Quantification of total RNA was performed on a Nanodrop spectrophotometer (ThermoFisher Scientific) and quality tested on an Agilent Tapestation with R6K Screentapes (RIN 7.6-9.8). Generation of sense-strand cDNA from purified total RNA followed by second-strand synthesis, *in vitro* transcription cRNA synthesis, single-stranded cDNA synthesis and RNA hydrolysis, fragmentation and labelling, was performed according to manufacturer's instructions (GeneChip WT Plus reagent kit, Affymetrix, Santa Clara, CA). Hybridisation, washing, staining and scanning of microarrays were carried out on Affymetrix Mouse Gene 2.0 ST microarrays using a GeneChip Fluidics station 450 and GCS3000 scanner (Affymetrix^®^, Santa Clara, CA).

Data pre-processing and quality control analysis was performed using Affymetrix^®^ Genechip^®^ Expression Console v1.2. Probe cell intensity data on the Mouse Gene 2.0 ST array (CEL files) were processed using the RMA16 algorithm (Affymetrix, Santa Clara, CA), which fits a robust linear model at probe level by employing background correction, quantile normalisation of log2-transformed data and summarisation to probe level data for primary quality control analysis. Experiments and analysis were performed in triplicate at Centre for Genome Enabled Biology and Medicine (University of Aberdeen, Aberdeen, UK). Microarray data is available via ArrayExpress accession E-MTAB-7614.

Data were analysed in Partek^®^ Genomics Suite^®^, version 6.6, build 6.15.0730 Copyright^©^; 2014 (Partek Inc., St Louis, MO) using a Mouse gene Gene 2.0 ST annotation file from build mm10, MoGene-2.0-st-v1.na35.mm10.transcript. Affymetrix CEL files were imported to Partek^®^ Genomics Suite^®^, and data processed using robust multiarray averaging (RMA) normalisation with RMA background correction and quantile normalisation of log2-transformed data and probeset summarisation by median polish. Two-way analysis of variance with time point (24 h, 48 h) and transcription factor (empty vector, VGLL3, YAP1 S127A, TAZ S89A) groups and time×transcription interaction was performed to evaluate significantly differentially expressed genes. YAP1 S127A and TAZ S89A (Sun et al., 2017) were included to allow comparison between VGLL3 and these Hippo pathway TEAD co-factors. The fold change for each gene in myoblasts expressing TAZ S89A, YAP S127A or VGLL3 compared to in myoblasts expressing empty vector, as a baseline, at each time point was calculated using the geometric mean of samples in each group with significance calculated by means of a Fisher's Least significant difference test. Genes with fold change of ≥1.3 fold and FDR of 10% in VGLL3 versus empty vector were further evaluated. The gene expression analysis was performed with the full set of samples (Empty Vector, VGLL3) and previously published YAP1 S127A and TAZ S89A results (Sun et al., 2017).

### Myofibre isolation and culture of mouse satellite cells

Mice aged between 8–12 weeks were killed by cervical dislocation and the extensor digitorum longus (EDL) muscles were isolated and digested as previously described (Judson et al., 2012). Freshly isolated myofibres were fixed in 4% PFA for 10 min, or plated on Matrigel, and the satellite cell-derived myoblasts were then expanded using DMEM-GlutaMAX (Invitrogen), with 30% FBS (Gibco), 10% horse serum (Invitrogen Life Technologies), 1% chick embryo extract (MP), 10 ng/ml bFGF (PreproTech) and 1% penicillin–streptomycin (Sigma), again as previously described (Moyle and Zammit, 2014).

### Isolation and culture of primary human myoblasts

Primary human myoblasts were obtained from biopsies of the vastus lateralis of consenting individuals [approved by the UK National Health Service Ethics Committee (London Research Ethics Committee; reference 10/H0718/10 and in accordance with the Human Tissue Act and Declaration of Helsinki)]. Biopsies were digested in basal medium (PromoCell containing collagenase B and dispase II) and single cells isolated via a 100 µm cell strainer as previously described (Agley et al., 2015). Adherent cells were cultured for 7 days in skeletal muscle cell growth medium (PromoCell, Heidelberg, Germany) and the NCAM/CD56^+^ myogenic population collected via magnetic-activated cell sorting (MACS).

### Retroviral expression vectors

The retroviral backbone *pMSCV-puro* (Clontech) was modified to replace the puromycin selection gene with an *IRES-eGFP* sequence to create *pMSCV-IRES-eGFP*, which served as control. Mouse *Vgll3* cDNA was amplified through a RT-PCR and cloned into *pMSCV-IRES-eGFP* to generate RV VGLL3. Retrovirus was then packaged in 293T cells using standard methods. Proliferating satellite cells were transduced in six-well plates with 500 μl of retrovirus in 1.5 ml of proliferation medium with polybrene (4 μg/ml).

Mouse *Vgll3* cDNA (transcript variant XM_006523098.2, which encodes a protein of 276 amino acids) was amplified by RT-PCR and cloned into *pMSCV-IRES-eGFP* to generate a 3xFlag-VGLL3 retrovirus. The human cDNA of *VGLL3* (transcript VGLL3-201, which encodes a protein of 320 amino acids) was cloned by RT-PCR from a plasmid provided by TransOMIC technologies (MGC premier cDNA clone for *VGLL3*: BC094780) into *pMSCV-IRES-eGFP* to generate RV VGLL3. Retrovirus (control; empty vector encoding only for GFP, and retrovirus containing VGLL3) were then packaged in Phoenix cells using standard methods. Proliferating immortalized human myoblasts (C25Cl48) were transduced in six-well plates with 200 μl of concentrated retrovirus in 1.8 ml of proliferation medium with polybrene (8 μg/ml). Cells were expanded and GFP-positive cells (transduced cells) were FAC sorted and expanded *in vitro* for a few days when stably transduced cells were analysed.

### siRNA-mediated gene knockdown

Transfection of siRNA into primary satellite cell-derived murine myoblasts from EDL myofibres was performed with Silencer select Pre-designed siRNA (Invitrogen Life Technologies) using Lipofectamine RNAiMAX. The siRNAs were: Ambion, siVGLL3 (silencer selected siRNA ID: s91841) and Silencer Select Negative Control #2 siRNA (4390847). Satellite cells were transfected with siRNA at a final concentration of 10 nM in DMEM-GlutaMAX, 30% FBS, 10% horse serum and 1% chick embryo extract for 24 h at 37°C. Transfection of siRNA into immortalized human myoblasts (C25Cl48) was performed with FlexiTube GeneSolution (four siRNAs targeting the gene of interest) (Qiagen) using Lipofectamine RNAiMAX. The siRNAs used against VGLL3 were Hs_VGLL3_1, SI03180310, Hs_VGLL3_3, SI03236233, Hs_VGLL3_4, SI04232312, Hs_VGLL3_5, SI104277819 (Qiagen) and the All Stars Negative control siRNA. Human myoblasts were transfected with a mix of 4 siRNA targeting VGLL3 and each individual siRNA used at a final concentration of 2 nM in skeletal muscle cell growth medium (Promo-Cell) for 24 h at 37°C.

### qRT-PCR

Primary satellite cell-derived murine myoblasts isolated from EDL muscle or primary human myoblasts obtained from biopsies of the vastus lateralis were cultured in six-well plates in proliferation medium or switched to differentiation medium for different periods of time (12, 24, 48 or 72 h). Total RNA was extracted using the RNeasy Kit (Qiagen) and cDNA prepared from 500 ng to 1 μg of RNA with the QuantiTect Reverse Transcription Kit with genomic DNA wipeout (Qiagen). qRT-PCR was performed on a Mx3005PQPCR system (Stratagene) with Brilliant II SYBR green reagents and ROX reference dye (Stratagene) using primers shown in Table S9. Relative expression between proliferating and differentiated myoblasts was measured in three replicates and significance was tested with a two-tailed Student's *t*-test using Microsoft Excel.

### Muscle cryosectioning and immunolabelling

EDL and Soleus muscles were harvested and frozen in liquid nitrogen-cooled isopentane. Frozen sections (10 µm) were cut on a cryostat, then dried and kept at −80°C before analysis (Ortuste Quiroga et al., 2016). Slides were allowed to warm up at room temperature, washed with PBS for 5 min and then blocked in 5% goat serum, 0.5% BSA and 0.2% triton X-100/PBS for 30 min. Primary antibodies (Table S8) were prepared in blocking solution and incubated overnight at 4°C. Sections were washed in 0.05% Tween 20/PBS and secondary antibodies (Molecular Probes; 1:500) were prepared in blocking solution and applied for 1 h at room temperature. Preparations were then washed in 0.05% Tween 20 (Merck) in PBS and then incubated in DAPI (300 µM) for 10 min, washed in PBS and mounted in Dako fluorescence mounting medium.

### Immunolabelling of cells

EdU incorporation was performed using a Click-iT EdU Imaging Kit (Invitrogen Life Technologies) as per the manufacturer's instructions. Primary murine satellite cell-derived myoblasts and C2C12 myoblasts were immunolabelled as previously described (Judson et al., 2012). Briefly, fixed myoblasts were permeabilised with 0.5% Triton X-100 in PBS for 5 min at room temperature and blocked with 5% goat serumin PBS for 60 min at room temperature. Primary antibodies [e.g. anti-Flag (F1804, Sigma), anti-MyHC (MF20, DSHB) and anti-Myogenin (F5D, DSHB); details are given in Table S1] were applied overnight at 4°C, and then cells were washed and visualised with fluorochrome-conjugated secondary antibodies (Invitrogen) used at 1:500 for 1 h at room temperature. Preparations were then incubated in DAPI (300 µM) for 10 min and washed in PBS.

### Image acquisition

Images of plated myoblasts and muscle cross-sections were acquired on a Zeiss Axiovert 200M microscope using a Zeiss AxioCam HRm and AxioVision software version 4.4 (Zeiss). Images were adjusted globally for brightness and contrast. Images of whole muscles were reconstructed from several pictures using Adobe Photoshop software (Fig. 6) and analysed (cell number counting, muscle fibre area and Ferret diameter) with ImageJ software.

### Statistical testing

Cells in multiple unit areas per experimental condition well were analysed, and all data from each of at least three mice or independent wells are expressed as a mean±s.e.m. A significant difference (*P*<0.05) between control and a test sample was determined using a two-tailed *t*-test in Excel (Microsoft).

## Supplementary Material

Supplementary information

## References

[JCS225946C1] Agley C. C., Rowlerson A. M., Velloso C. P., Lazarus N. L. and Harridge S. D. R. (2015). Isolation and quantitative immunocytochemical characterization of primary myogenic cells and fibroblasts from human skeletal muscle. *J. Vis. Exp.*, 95, e52049 10.3791/52049 PMC4354531 25650991

[JCS225946C2] Akaike K., Suehara Y., Kohsaka S., Hayashi T., Tanabe Y., Kazuno S., Mukaihara K., Toda-Ishii M., Kurihara T., Kim Y. et al. (2018). PPP2R1A regulated by PAX3/FOXO1 fusion contributes to the acquisition of aggressive behavior in PAX3/FOXO1-positive alveolar rhabdomyosarcoma. *Oncotarget* 9, 25206-25215. 10.18632/oncotarget.25392 29861864 PMC5982774

[JCS225946C3] Allard J. B. and Duan C. (2018). IGF-binding proteins: why do they exist and why are there so many? *Front. Endocrinol. (Lausanne)* 9, e117. 10.3389/fendo.2018.00117 PMC5900387 29686648

[JCS225946C4] Benhaddou A., Keime C., Ye T., Morlon A., Michel I., Jost B., Mengus G. and Davidson I. (2012). Transcription factor TEAD4 regulates expression of myogenin and the unfolded protein response genes during C2C12 cell differentiation. *Cell Death Differ.* 19, 220-231. 10.1038/cdd.2011.87 21701496 PMC3263497

[JCS225946C5] Bernard F., Kasherov P., Grenetier S., Dutriaux A., Zider A., Silber J. and Lalouette A. (2009). Integration of differentiation signals during indirect flight muscle formation by a novel enhancer of Drosophila vestigial gene. *Dev. Biol.* 332, 258-272. 10.1016/j.ydbio.2009.05.573 19500564

[JCS225946C6] Bertrand A. T., Ziaei S., Ehret C., Duchemin H., Mamchaoui K., Bigot A., Mayer M., Quijano-Roy S., Desguerre I., Laine J. et al. (2014). Cellular microenvironments reveal defective mechanosensing responses and elevated YAP signaling in LMNA-mutated muscle precursors. *J. Cell Sci.* 127, 2873-2884. 10.1242/jcs.144907 24806962

[JCS225946C7] Brown S. D. M. and Moore M. W. (2012). The International Mouse Phenotyping Consortium: past and future perspectives on mouse phenotyping. *Mamm. Genome* 23, 632-640. 10.1007/s00335-012-9427-x 22940749 PMC3774932

[JCS225946C8] Cancer Genome Atlas Research Network. (2017). Comprehensive and Integrated Genomic Characterization of Adult Soft Tissue Sarcomas. *Cell* 171, 950-965.e28. 10.1016/j.cell.2017.10.014 29100075 PMC5693358

[JCS225946C9] Cao L., Yu Y., Bilke S., Walker R. L., Mayeenuddin L. H., Azorsa D. O., Yang F., Pineda M., Helman L. J. and Meltzer P. S. (2010). Genome-wide identification of PAX3-FKHR binding sites in rhabdomyosarcoma reveals candidate target genes important for development and cancer. *Cancer Res.* 70, 6497-6508. 10.1158/0008-5472.CAN-10-0582 20663909 PMC2922412

[JCS225946C10] Chaillou T., Lee J. D., England J. H., Esser K. A. and McCarthy J. J. (2013). Time course of gene expression during mouse skeletal muscle hypertrophy. *J. Appl. Physiol.* 115, 1065-1074. 10.1152/japplphysiol.00611.2013 23869057 PMC3798821

[JCS225946C11] Chen H.-H., Maeda T., Mullett S. J. and Stewart A. F. R. (2004). Transcription cofactor Vgl-2 is required for skeletal muscle differentiation. *Genesis* 39, 273-279. 10.1002/gene.20055 15287000

[JCS225946C12] Chen Q., Zhang N., Xie R., Wang W., Cai J., Choi K.-S., David K. K., Huang B., Yabuta N., Nojima H. et al. (2015). Homeostatic control of Hippo signaling activity revealed by an endogenous activating mutation in YAP. *Genes Dev.* 29, 1285-1297. 10.1101/gad.264234.115 26109051 PMC4495399

[JCS225946C13] Davicioni E., Anderson M. J., Finckenstein F. G., Lynch J. C., Qualman S. J., Shimada H., Schofield D. E., Buckley J. D., Meyer W. H., Sorensen P. H. B. et al. (2009). Molecular classification of rhabdomyosarcoma—genotypic and phenotypic determinants of diagnosis: a report from the Children's Oncology Group. *Am. J. Pathol.* 174, 550-564. 10.2353/ajpath.2009.080631 19147825 PMC2630563

[JCS225946C14] G-TEx-Consortium. (2015). Human genomics. The Genotype-Tissue Expression (GTEx) pilot analysis: multitissue gene regulation in humans. *Science* 348, 648-660. 10.1126/science.1262110 25954001 PMC4547484

[JCS225946C15] Gabriel B. M., Hamilton D. L., Tremblay A. M. and Wackerhage H. (2016). The Hippo signal transduction network for exercise physiologists. *J. Appl. Physiol.* 120, 1105-1117. 10.1152/japplphysiol.01076.2015 26940657 PMC4867322

[JCS225946C16] Gambaro K., Quinn M. C. J., Wojnarowicz P. M., Arcand S. L., de Ladurantaye M., Barrès V., Ripeau J.-S., Killary A. M., Davis E. C., Lavoie J. et al. (2013). VGLL3 expression is associated with a tumor suppressor phenotype in epithelial ovarian cancer. *Mol. Oncol.* 7, 513-530. 10.1016/j.molonc.2012.12.006 23415753 PMC5528482

[JCS225946C17] Ganti R., Skapek S. X., Zhang J., Fuller C. E., Wu J., Billups C. A., Breitfeld P. P., Dalton J. D., Meyer W. H. and Khoury J. D. (2006). Expression and genomic status of EGFR and ErbB-2 in alveolar and embryonal rhabdomyosarcoma. *Mod. Pathol.* 19, 1213-1220. 10.1038/modpathol.3800636 16729016

[JCS225946C18] Goodman C. A., Dietz J. M., Jacobs B. L., McNally R. M., You J.-S. and Hornberger T. A. (2015). Yes-Associated Protein is up-regulated by mechanical overload and is sufficient to induce skeletal muscle hypertrophy. *FEBS Lett.* 589, 1491-1497. 10.1016/j.febslet.2015.04.047 25959868 PMC4442043

[JCS225946C19] Gunther S., Mielcarek M., Kruger M. and Braun T. (2004). VITO-1 is an essential cofactor of TEF1-dependent muscle-specific gene regulation. *Nucleic Acids Res.* 32, 791-802. 10.1093/nar/gkh248 14762206 PMC373362

[JCS225946C20] Halder G. and Carroll S. B. (2001). Binding of the Vestigial co-factor switches the DNA-target selectivity of the Scalloped selector protein. *Development* 128, 3295-3305. 11546746 10.1242/dev.128.17.3295

[JCS225946C21] Halder G., Polaczyk P., Kraus M. E., Hudson A., Kim J., Laughon A. and Carroll S. (1998). The Vestigial and Scalloped proteins act together to directly regulate wing-specific gene expression in Drosophila. *Genes Dev.* 12, 3900-3909. 10.1101/gad.12.24.3900 9869643 PMC317267

[JCS225946C22] Hansen C. G., Moroishi T. and Guan K.-L. (2015). YAP and TAZ: a nexus for Hippo signaling and beyond. *Trends Cell Biol.* 25, 499-513. 10.1016/j.tcb.2015.05.002 26045258 PMC4554827

[JCS225946C23] Haslett J. N., Sanoudou D., Kho A. T., Han M., Bennett R. R., Kohane I. S., Beggs A. H. and Kunkel L. M. (2003). Gene expression profiling of Duchenne muscular dystrophy skeletal muscle. *Neurogenetics* 4, 163-171. 10.1007/s10048-003-0148-x 12698323

[JCS225946C24] Hélias-Rodzewicz Z., Pérot G., Chibon F., Ferreira C., Lagarde P., Terrier P., Coindre J.-M. and Aurias A. (2010). YAP1 and VGLL3, encoding two cofactors of TEAD transcription factors, are amplified and overexpressed in a subset of soft tissue sarcomas. *Genes Chromosomes Cancer* 49, 1161-1171. 10.1002/gcc.20825 20842732

[JCS225946C25] Hoffman N. J., Parker B. L., Chaudhuri R., Fisher-Wellman K. H., Kleinert M., Humphrey S. J., Yang P., Holliday M., Trefely S., Fazakerley D. J. et al. (2015). Global phosphoproteomic analysis of human skeletal muscle reveals a network of exercise-regulated kinases and AMPK substrates. *Cell Metab.* 22, 922-935. 10.1016/j.cmet.2015.09.001 26437602 PMC4635038

[JCS225946C26] Honda M., Hidaka K., Fukada S.-I., Sugawa R., Shirai M., Ikawa M. and Morisaki T. (2017). Vestigial-like 2 contributes to normal muscle fiber type distribution in mice. *Sci. Rep.* 7, 7168 10.1038/s41598-017-07149-0 28769032 PMC5540913

[JCS225946C27] Jiao S., Wang H., Shi Z., Dong A., Zhang W., Song X., He F., Wang Y., Zhang Z., Wang W. et al. (2014). A peptide mimicking VGLL4 function acts as a YAP antagonist therapy against gastric cancer. *Cancer Cell* 25, 166-180. 10.1016/j.ccr.2014.01.010 24525233

[JCS225946C28] Judson R. N., Tremblay A. M., Knopp P., White R. B., Urcia R., De Bari C., Zammit P. S., Camargo F. D. and Wackerhage H. (2012). The Hippo pathway member YAP plays a key role in influencing fate decisions in muscle satellite cells. *J. Cell Sci.* 125, 6009-6019. 10.1242/jcs.109546 23038772 PMC3585517

[JCS225946C29] Judson R. N., Gray S. R., Walker C., Carroll A. M., Itzstein C., Lionikas A., Zammit P. S., De Bari C. and Wackerhage H. (2013). Constitutive expression of Yes-associated protein (YAP) in adult skeletal muscle fibres induces muscle atrophy and myopathy. *PLoS ONE* 8, e59622 10.1371/journal.pone.0059622 23544078 PMC3609830

[JCS225946C30] Kassar-Duchossoy L., Gayraud-Morel B., Gomès D., Rocancourt D., Buckingham M., Shinin V. and Tajbakhsh S. (2004). Mrf4 determines skeletal muscle identity in Myf5:Myod double-mutant mice. *Nature* 431, 466-471. 10.1038/nature02876 15386014

[JCS225946C31] Kelley L. A., Mezulis S., Yates C. M., Wass M. N. and Sternberg M. J. E. (2015). The Phyre2 web portal for protein modeling, prediction and analysis. *Nat. Protoc.* 10, 845-858. 10.1038/nprot.2015.053 25950237 PMC5298202

[JCS225946C32] Knopp P., Figeac N., Fortier M., Moyle L. and Zammit P. S. (2013). Pitx genes are redeployed in adult myogenesis where they can act to promote myogenic differentiation in muscle satellite cells. *Dev. Biol.* 377, 293-304. 10.1016/j.ydbio.2013.02.011 23438814

[JCS225946C33] Koontz L. M., Liu-Chittenden Y., Yin F., Zheng Y., Yu J., Huang B., Chen Q., Wu S. and Pan D. (2013). The Hippo effector Yorkie controls normal tissue growth by antagonizing scalloped-mediated default repression. *Dev. Cell* 25, 388-401. 10.1016/j.devcel.2013.04.021 23725764 PMC3705890

[JCS225946C34] Lagha M., Sato T., Regnault B., Cumano A., Zuniga A., Licht J., Relaix F. and Buckingham M. (2010). Transcriptome analyses based on genetic screens for Pax3 myogenic targets in the mouse embryo. *BMC.Genomics* 11, 696 10.1186/1471-2164-11-696 21143873 PMC3018477

[JCS225946C35] L'Honore A., Commère P.-H., Ouimette J.-F., Montarras D., Drouin J. and Buckingham M. (2014). Redox regulation by Pitx2 and Pitx3 is critical for fetal myogenesis. *Dev. Cell* 29, 392-405. 10.1016/j.devcel.2014.04.006 24871946

[JCS225946C36] Liang Y., Tsoi L. C., Xing X., Beamer M. A., Swindell W. R., Sarkar M. K., Berthier C. C., Stuart P. E., Harms P. W., Nair R. P. et al. (2017). A gene network regulated by the transcription factor VGLL3 as a promoter of sex-biased autoimmune diseases. *Nat. Immunol.* 18, 152-160. 10.1038/ni.3643 27992404 PMC5289297

[JCS225946C37] Lukjanenko L., Brachat S., Pierrel E., Lach-Trifilieff E. and Feige J. N. (2013). Genomic profiling reveals that transient adipogenic activation is a hallmark of mouse models of skeletal muscle regeneration. *PLoS ONE* 8, e71084 10.1371/journal.pone.0071084 23976982 PMC3744575

[JCS225946C38] Maeda T., Chapman D. L. and Stewart A. F. R. (2002). Mammalian vestigial-like 2, a cofactor of TEF-1 and MEF2 transcription factors that promotes skeletal muscle differentiation. *J. Biol. Chem.* 277, 48889-48898. 10.1074/jbc.M206858200 12376544

[JCS225946C39] Mamchaoui K., Trollet C., Bigot A., Negroni E., Chaouch S., Wolff A., Kandalla P. K., Marie S., Di Santo J., St Guily J. L. et al. (2011). Immortalized pathological human myoblasts: towards a universal tool for the study of neuromuscular disorders. *Skelet. Muscle* 1, 34 10.1186/2044-5040-1-34 22040608 PMC3235972

[JCS225946C40] Mielcarek M., Günther S., Krüger M. and Braun T. (2002). VITO-1, a novel vestigial related protein is predominantly expressed in the skeletal muscle lineage. *Mech. Dev.* 119 Suppl. 1, S269-S274. 10.1016/S0925-4773(03)00127-8 14516696

[JCS225946C41] Mielcarek M., Piotrowska I., Schneider A., Günther S. and Braun T. (2009). VITO-2, a new SID domain protein, is expressed in the myogenic lineage during early mouse embryonic development. *Gene Expr. Patterns* 9, 129-137. 10.1016/j.gep.2008.12.002 19118645

[JCS225946C42] Missiaglia E., Williamson D., Chisholm J., Wirapati P., Pierron G., Petel F., Concordet J.-P., Thway K., Oberlin O., Pritchard-Jones K. et al. (2012). PAX3/FOXO1 fusion gene status is the key prognostic molecular marker in rhabdomyosarcoma and significantly improves current risk stratification. *J. Clin. Oncol.* 30, 1670-1677. 10.1200/JCO.2011.38.5591 22454413

[JCS225946C43] Mohamed A., Sun C., De Mello V., Selfe J., Missiaglia E., Shipley J., Murray G. I., Zammit P. S. and Wackerhage H. (2016). The Hippo effector TAZ (WWTR1) transforms myoblasts and TAZ abundance is associated with reduced survival in embryonal rhabdomyosarcoma. *J. Pathol.* 240, 3-14. 10.1002/path.4745 27184927 PMC4995731

[JCS225946C44] Moyle L. A. and Zammit P. S. (2014). Isolation, culture and immunostaining of skeletal muscle fibres to study myogenic progression in satellite cells. *Methods Mol. Biol.* 1210, 63-78. 10.1007/978-1-4939-1435-7_6 25173161

[JCS225946C45] Ortuste Quiroga H. P., Goto K. and Zammit P. S. (2016). Isolation, Cryosection and Immunostaining of Skeletal Muscle. *Methods Mol. Biol.* 1460, 85-100. 10.1007/978-1-4939-3810-0_8 27492168

[JCS225946C46] Ouchi N., Oshima Y., Ohashi K., Higuchi A., Ikegami C., Izumiya Y. and Walsh K. (2008). Follistatin-like 1, a secreted muscle protein, promotes endothelial cell function and revascularization in ischemic tissue through a nitric-oxide synthase-dependent mechanism. *J. Biol. Chem.* 283, 32802-32811. 10.1074/jbc.M803440200 18718903 PMC2583310

[JCS225946C47] Park H. W., Kim Y. C., Yu B., Moroishi T., Mo J.-S., Plouffe S. W., Meng Z., Lin K. C., Yu F.-X., Alexander C. M. et al. (2015). Alternative Wnt signaling activates YAP/TAZ. *Cell* 162, 780-794. 10.1016/j.cell.2015.07.013 26276632 PMC4538707

[JCS225946C48] Perez-Riverol Y., Alpi E., Wang R., Hermjakob H. and Vizcaíno J. A. (2015). Making proteomics data accessible and reusable: current state of proteomics databases and repositories. *Proteomics* 15, 930-949. 10.1002/pmic.201400302 25158685 PMC4409848

[JCS225946C49] Piccolo S., Dupont S. and Cordenonsi M. (2014). The biology of YAP/TAZ: hippo signaling and beyond. *Physiol. Rev.* 94, 1287-1312. 10.1152/physrev.00005.2014 25287865

[JCS225946C50] Pimmett V. L., Deng H., Haskins J. A., Mercier R. J., LaPointe P. and Simmonds A. J. (2017). The activity of the Drosophila Vestigial protein is modified by Scalloped-dependent phosphorylation. *Dev. Biol.* 425, 58-69. 10.1016/j.ydbio.2017.03.013 28322734

[JCS225946C51] Pobbati A. V., Chan S. W., Lee I., Song H. and Hong W. (2012). Structural and functional similarity between the VGLL1-TEAD and the YAP-TEAD complexes. *Structure* 20, 1135-1140. 10.1016/j.str.2012.04.004 22632831

[JCS225946C52] Potts G. K., McNally R. M., Blanco R., You J.-S., Hebert A. S., Westphall M. S., Coon J. J. and Hornberger T. A. (2017). A map of the phosphoproteomic alterations that occur after a bout of maximal-intensity contractions. *J. Physiol.* 595, 5209-5226. 10.1113/JP273904 28542873 PMC5538225

[JCS225946C53] Ribas R., Moncaut N., Siligan C., Taylor K., Cross J. W., Rigby P. W. J. and Carvajal J. J. (2011). Members of the TEAD family of transcription factors regulate the expression of Myf5 in ventral somitic compartments. *Dev. Biol.* 355, 372-380. 10.1016/j.ydbio.2011.04.005 21527258 PMC3123743

[JCS225946C54] Rudnicki M. A., Schnegelsberg P. N. J., STEAD R. H., Braun T., Arnold H.-H. and Jaenisch R. (1993). MyoD or Myf-5 is required for the formation of skeletal muscle. *Cell* 75, 1351-1359. 10.1016/0092-8674(93)90621-V 8269513

[JCS225946C55] Shern J. F., Chen L., Chmielecki J., Wei J. S., Patidar R., Rosenberg M., Ambrogio L., Auclair D., Wang J., Song Y. K. et al. (2014). Comprehensive genomic analysis of rhabdomyosarcoma reveals a landscape of alterations affecting a common genetic axis in fusion-positive and fusion-negative tumors. *Cancer Discov.* 4, 216-231. 10.1158/2159-8290.CD-13-0639 24436047 PMC4462130

[JCS225946C56] Simon E., Faucheux C., Zider A., Thézé N. and Thiébaud P. (2016). From vestigial to vestigial-like: the Drosophila gene that has taken wing. *Dev. Genes Evol.* 226, 297-315. 10.1007/s00427-016-0546-3 27116603

[JCS225946C57] Simon E., Thézé N., Fédou S., Thiébaud P. and Faucheux C. (2017). Vestigial-like 3 is a novel Ets1 interacting partner and regulates trigeminal nerve formation and cranial neural crest migration. *Biol. Open* 6, 1528-1540. 10.1242/bio.026153 28870996 PMC5665465

[JCS225946C58] Slemmons K. K., Crose L. E. S., Rudzinski E., Bentley R. C. and Linardic C. M. (2015). Role of the YAP oncoprotein in priming Ras-driven rhabdomyosarcoma. *PLoS ONE* 10, e0140781 10.1371/journal.pone.0140781 26496700 PMC4619859

[JCS225946C59] Sun C., De Mello V., Mohamed A., Ortuste Quiroga H. P., Garcia-Munoz A., Al Bloshi A., Tremblay A. M., von Kriegsheim A., Collie-Duguid E., Vargesson N. et al. (2017). Common and distinctive functions of the Hippo effectors TAZ and YAP in skeletal muscle stem cell function. *Stem Cells* 35, 1958-1972. 10.1002/stem.2652 28589555 PMC5575518

[JCS225946C60] Tremblay A. M. and Camargo F. D. (2012). Hippo signaling in mammalian stem cells. *Semin. Cell Dev. Biol.* 23, 818-826. 10.1016/j.semcdb.2012.08.001 23034192

[JCS225946C61] Tremblay A. M., Missiaglia E., Galli G. G., Hettmer S., Urcia R., Carrara M., Judson R. N., Thway K., Nadal G., Selfe J. L. et al. (2014). The Hippo transducer YAP1 transforms activated satellite cells and is a potent effector of embryonal rhabdomyosarcoma formation. *Cancer Cell* 26, 273-287. 10.1016/j.ccr.2014.05.029 25087979

[JCS225946C62] Tsika R. W., Schramm C., Simmer G., Fitzsimons D. P., Moss R. L. and Ji J. (2008). Overexpression of TEAD-1 in transgenic mouse striated muscles produces a slower skeletal muscle contractile phenotype. *J. Biol. Chem.* 283, 36154-36167. 10.1074/jbc.M807461200 18978355 PMC2606011

[JCS225946C63] Turriziani B., Garcia-Munoz A., Pilkington R., Raso C., Kolch W. and von Kriegsheim A. (2014). On-beads digestion in conjunction with data-dependent mass spectrometry: a shortcut to quantitative and dynamic interaction proteomics. *Biology (Basel)* 3, 320-332. 10.3390/biology3020320 24833512 PMC4085610

[JCS225946C64] Vaudin P., Delanoue R., Davidson I., Silber J. and Zider A. (1999). TONDU (TDU), a novel human protein related to the product of vestigial (vg) gene of Drosophila melanogaster interacts with vertebrate TEF factors and substitutes for Vg function in wing formation. *Development* 126, 4807-4816. 10518497 10.1242/dev.126.21.4807

[JCS225946C65] Vissing K. and Schjerling P. (2014). Simplified data access on human skeletal muscle transcriptome responses to differentiated exercise. *Scientific data* 1, 140041 10.1038/sdata.2014.41 25984345 PMC4432635

[JCS225946C66] Vizcaíno J. A., Csordas A., Del-Toro N., Dianes J. A., Griss J., Lavidas I., Mayer G., Perez-Riverol Y., Reisinger F., Ternent T. et al. (2016). 2016 update of the PRIDE database and its related tools. *Nucleic Acids Res.* 44, 11033 10.1093/nar/gkw880 27683222 PMC5159556

[JCS225946C67] Wackerhage H., Del Re D. P., Judson R. N., Sudol M. and Sadoshima J. (2014). The Hippo signal transduction network in skeletal and cardiac muscle. *Sci. Signal.* 7, re4 10.1126/scisignal.2005096 25097035

[JCS225946C68] Wang P., Bouwman F. G. and Mariman E. C. M. (2009). Generally detected proteins in comparative proteomics—a matter of cellular stress response? *Proteomics* 9, 2955-2966. 10.1002/pmic.200800826 19415655

[JCS225946C69] Watt K. I., Judson R., Medlow P., Reid K., Kurth T. B., Burniston J. G., Ratkevicius A., De Bari C. and Wackerhage H. (2010). YAP is a novel regulator of C2C12 myogenesis. *Biochem. Biophys. Res. Commun.* 393, 619-624. 10.1016/j.bbrc.2010.02.034 20153295

[JCS225946C70] Watt K. I., Turner B. J., Hagg A., Zhang X., Davey J. R., Qian H., Beyer C., Winbanks C. E., Harvey K. F. and Gregorevic P. (2015). The Hippo pathway effector YAP is a critical regulator of skeletal muscle fibre size. *Nat. Commun.* 6, 6048 10.1038/ncomms7048 25581281

[JCS225946C71] Williamson D., Missiaglia E., de Reyniès A., Pierron G., Thuille B., Palenzuela G., Thway K., Orbach D., Laé M., Fréneaux P. et al. (2010). Fusion gene-negative alveolar rhabdomyosarcoma is clinically and molecularly indistinguishable from embryonal rhabdomyosarcoma. *J. Clin. Oncol.* 28, 2151-2158. 10.1200/JCO.2009.26.3814 20351326

