# Supplementary material for: VGLL3 operates via TEAD1, TEAD3 and TEAD4 to influence myogenesis in skeletal muscle
Source: J Cell Sci. 2019 Jul 5;132(13):jcs225946. doi: 10.1242/jcs.225946 (PMC6633393; doi:10.1242/jcs.225946)
Supplement: Supplementary information [file joces-132-225946-s1.pdf]

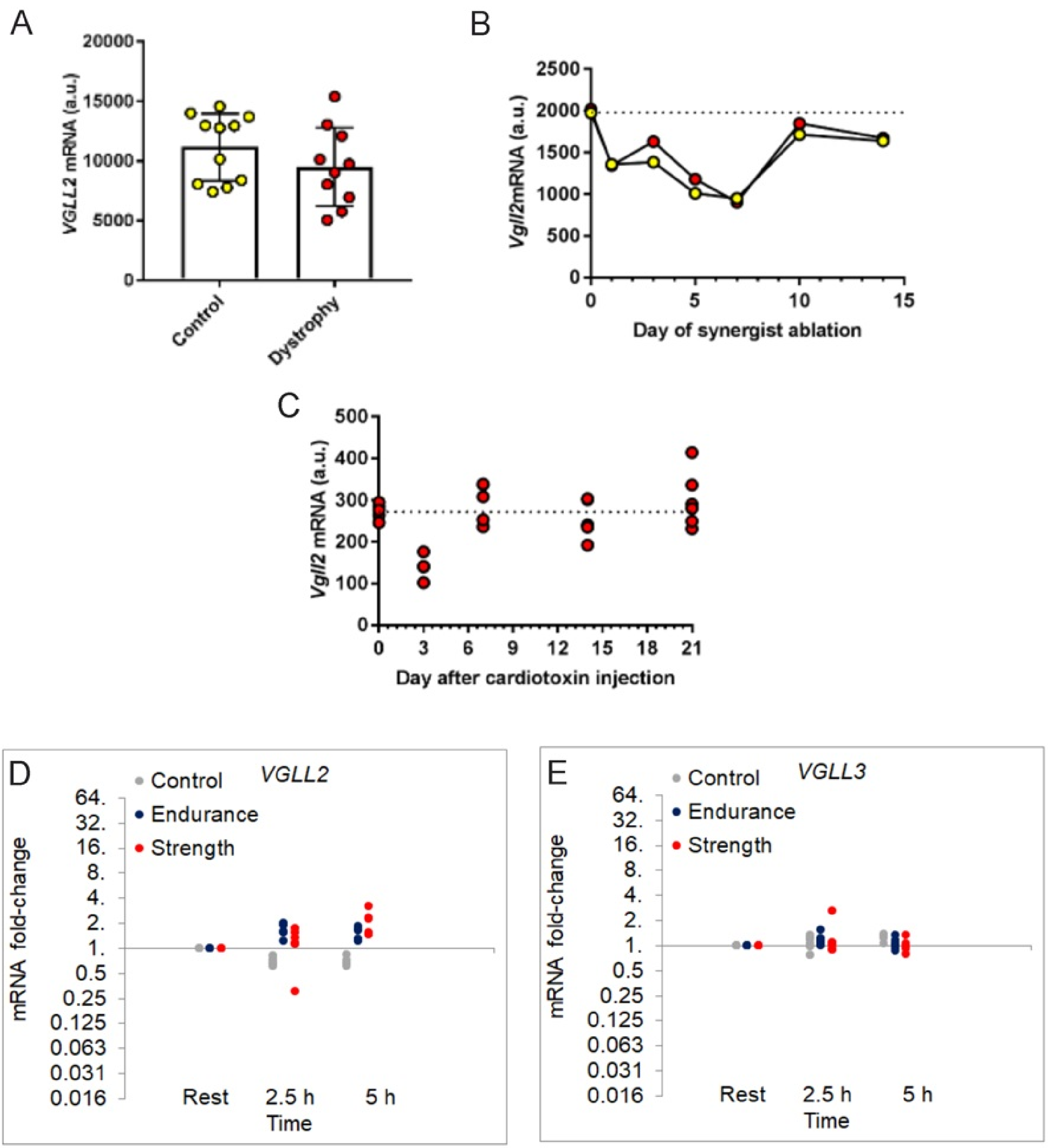

Figure S1: Expression of *Vgll2* and *Vgll3* in muscle

(A) VGLL2 expression in the quadriceps muscle of boys with Duchenne muscular dystrophy versus healthy controls (Retrieval of gene expression data from: **Haslett, J. N., Sanoudou, D., Kho, A. T., Han, M., Bennett, R. R., Kohane, I. S., Beggs, A. H. and Kunkel, L. M.** (2003). Gene expression profiling of Duchenne muscular dystrophy skeletal muscle. *Neurogenetics* **4**, 163-71).
(B) Vgll2 expression in mouse plantaris muscle overloaded through synergist ablation starting at day 0 (Retrieval of gene expression data from: **Chaillou, T., Lee, J. D., England, J. H., Esser, K. A. and McCarthy, J. J.** (2013). Time course of gene expression during mouse skeletal muscle hypertrophy. *J Appl Physiol (1985)* **115**, 1065-74).
(C) Vgll2 expression in mouse tibialis anterior muscle injured with cardiotoxin injection at day 0 (Retrieval of gene expression data from: **Lukjanenko, L., Brachat, S., Pierrel, E., Lach-Trifilieff, E. and Feige, J. N.** (2013). Genomic profiling reveals that transient adipogenic activation is a hallmark of mouse models of skeletal muscle regeneration. *PLoS ONE* **8**, e71084).
(D) *VGLL2* and **(E)** *VGLL3* expression in the vastus lateralis muscle before, 2.5 and 5 h after endurance or resistance exercise (Retrieval of gene expression data from: **Vissing, K. and Schjerling, P.** (2014). Simplified data access on human skeletal muscle transcriptome responses to differentiated exercise. *Sci Data* **1**, 140041).

Journal of Cell Science • Supplementary information

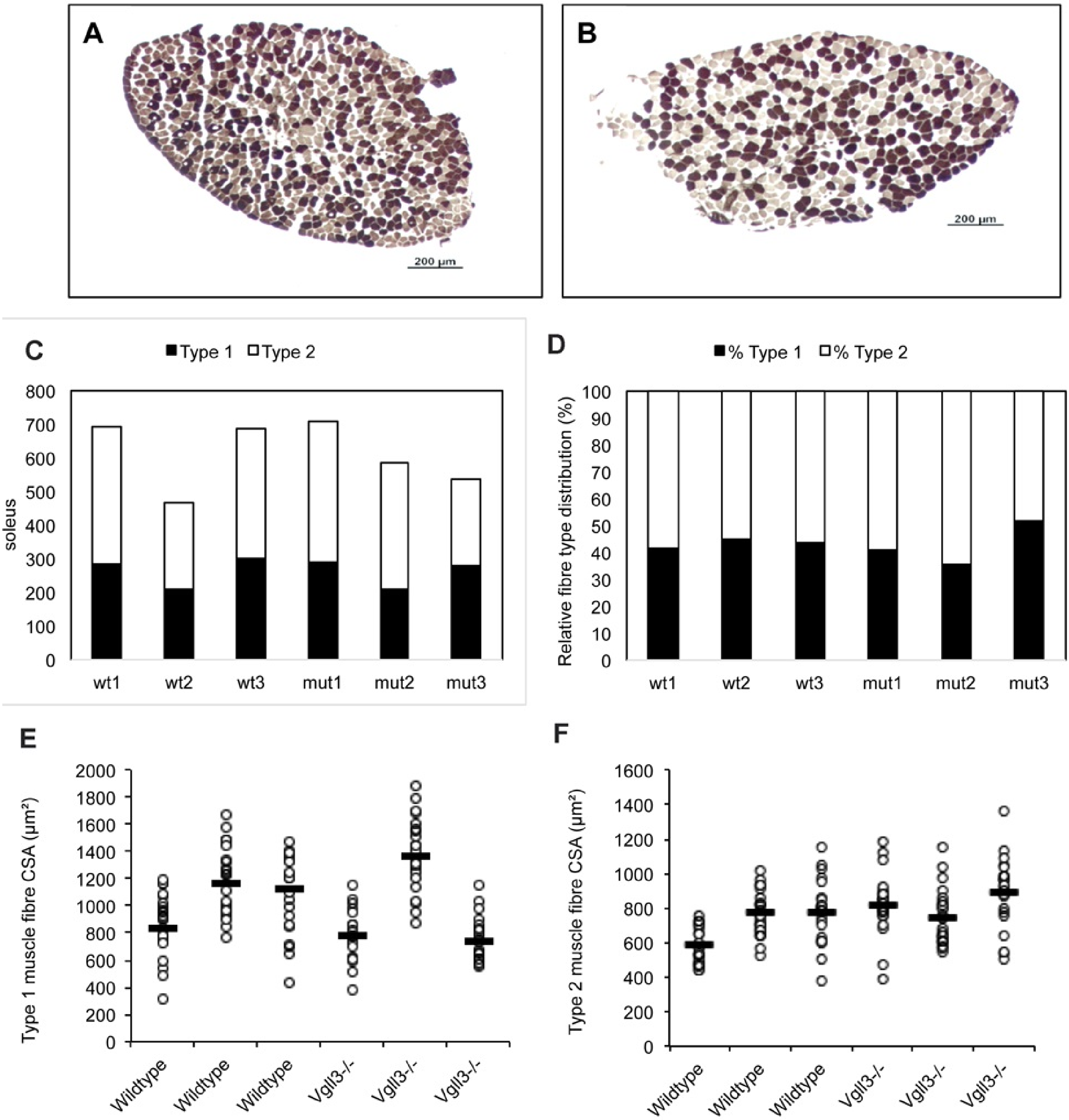

Figure S2: Vgll3 knockout does not affect muscle fibre type distribution or myofibre cross-sectional area in hind limb muscles
(**A-B**) ATPase-stained section of a wildtype soleus (**A**) or the soleus from a *Vgll3* knock out mouse (**B**).
(**C**) Absolute and (**D**) relative type 1 and type 2 (2a) fibre numbers in the soleus of wildtype (wt) and *Vgll3*$^{-/-}$(mut) mice.
(**E-F**) Type 1 and type 2 (2a) muscle fibre cross sectional area (CSA) of the gastrocnemius of wildtype and *Vgll3*$^{-/-}$ mice.

Journal of Cell Science • Supplementary information

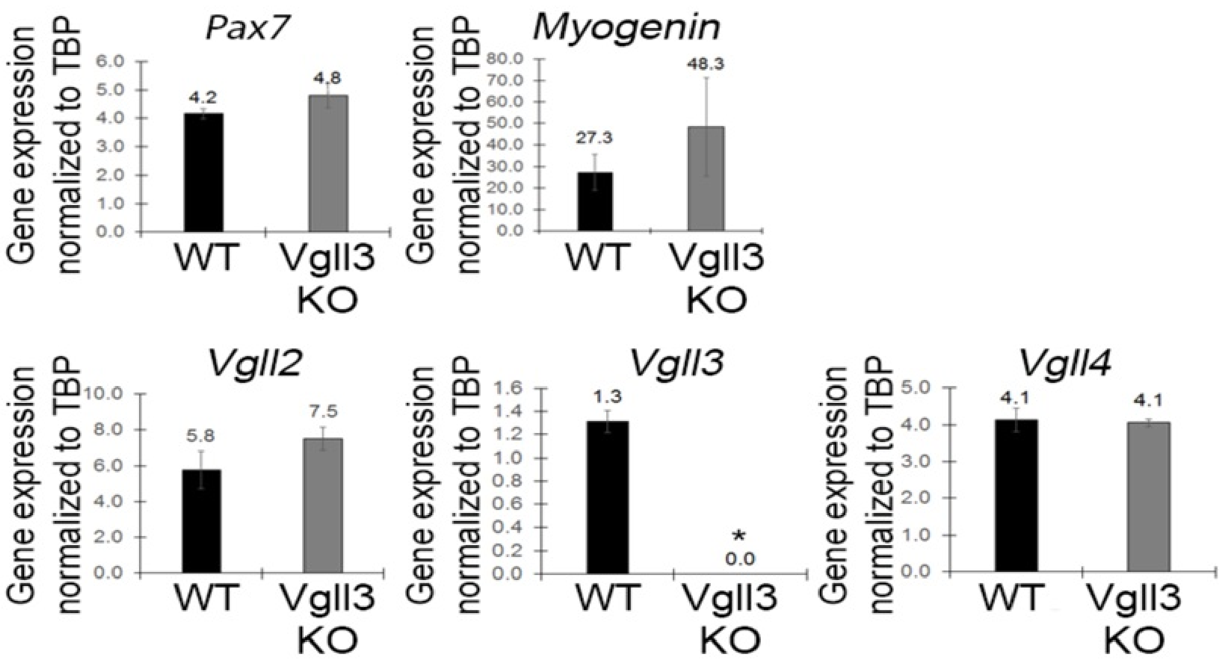

Figure S3: Vgll3 KO-derived satellite cells have unchanged gene expression
Expression of *Pax7*, *Myogenin*, *Vgll2*, *Vgll3* and *Vgll4* were analysed by RT-qPCR in proliferating satellite cells isolated from WT and *Vgll3*$^{-/-}$ (KO) mice and found not to be significantly different. Data are presented as mean ± SEM, where an asterisk would indicate a significant difference (p<0.05) between a test sample and control using a unpaired two-tailed t-test with n=3 mice.

Table S1: Proteins that bind VGLL3 in the skeletal muscle lineage
Downstream, VGLL3 binds TEAD1, TEAD3 and TEAD4 in C2C12 cells. Upstream, the key protein groups are Heat shock and related proteins, tubulins, metabolic genes and mitochondrial channels. VGLL3 targets TEAD1,3,4 transcription factors and no other transcription factors in the muscle lineage under standard cell culture conditions. The function of the upstream binding proteins is unknown.

Click here to Download Table S1

Table S2: Proteins that bind VGLL3, YAP and TAZ in C2C12 myoblasts and myotubes
(**A**) Comparison of VGLL3 with YAP and TAZ binding partners as we described in Sun, C., De Mello, V., Mohamed, A., Ortuste Quiroga, H. P., Garcia-Munoz, A., Al Bloshi, A., Tremblay, A. M., von Kriegsheim, A., Collie-Duguid, E., Vargesson, N. et al. (2017). Common and Distinctive Functions of the Hippo Effectors Taz and Yap in Skeletal Muscle Stem Cell Function. Stem Cells 35, 1958-1972. VGLL3-flag, YAP-flag and TAZ-flag all bind Tead1,3,4 in C2C12 cells.
(**B**) Whilst there is an 18.6% overlap between YAP-flag and TAZ-flag binding partners, the overlap with VGLL3-flag binding partners is only ~1%. Only 4 proteins (1.3%) bind both VGLL3-flag and YAP-flag and 3 proteins (1%) bind both VGLL3-flag and YAP-flag. Collectively this suggest that VGLL3, YAP and TAZ all target TEAD family transcription factors in C2C12 myoblasts and myotubes. However, non-transcription factor-binding partners are largely different.

Click here to Download Table S2

Table S3: Analysis of the human VGLL3 FASTA sequence led to the prediction of a MQDSLEVTL nuclear export signal which was fully conserved between man, chimpanzee, cat and mouse
(**A**) Human VGLL3 FASTA sequence from Swissprot and used NetNES 1.1 (http://www.cbs.dtu.dk/services/NetNES/) to predict nuclear export signals (la Cour, T., Kiemer, L., Molgaard, A., Gupta, R., Skriver, K. and Brunak, S. (2004). Analysis and prediction of leucine-rich nuclear export signals. Protein Eng Des Sel 17, 527-36).
(**B**) Evolutionary conservation of the human, chimpanzee, cat and mouse Vgll3 protein sequences using Clustal Omega (https://www.ebi.ac.uk/Tools/msa/clustalo/).

Click here to Download Table S3

Table S4: Genes that are up- or down-regulated by 24 h or 48 h of Vgll3 expression in murine primary satellite cell-derived myoblasts
(A) Genes that are significantly 1.3-fold up or 1.3-fold down (indicated with a "-") regulated at 24 h.
(B) Genes that are significantly 1.3-fold up or 1.3-fold down (indicated with a "-") regulated at 48 h.
Gene expression analysis using Affymetrix Mouse Gene 2.0 ST microarrays. Vgll3 is mainly a repressor of gene expression (ratio 9 down versus 1 up at 24 h and 29 up versus 126 down at 48 h).
Key: Vgll2 regulated genes are other Tead1-4 binding proteins (Vgll2, Wwtr1), the Hippo negative feedback loop (Ajuba, Amotl2, Frmd6), the myogenic regulator factor Myf5, insulin-like growth factor-binding proteins (Igfbp2-4) and Wnt proteins (Wnt7b, Fzd4) as well as Pitx transcription factors (Pitx2, Pitx3). The effect on Hippo signalling proteins is typical for the effect of Hippo proteins on transcription, suggesting that Vgll3 partially regulates Hippo target genes.

Click here to Download Table S4

Journal of Cell Science • Supplementary information

Table S5: Comparison of genes regulated by Vgll3 compared to Yap and Taz

Expression of either Vgll3, YAP1 S127A or TAZ S89A or empty vector for 24 h and 48 h in satellite cell-derived myoblasts. Gene expression analysis using Affymetrix Mouse Gene 2.0 ST microarrays. To avoid having to compare 6 datasets (Vgll3, Yap, Taz at 24 h and 48 h timepoints) we pooled the 24 h and 48 h time points. Vgll3 is mainly a repressor of gene expression and typically a Yap antagonist (examples: *Gzmd, Thbs1*) or co-represses genes with Yap (examples: *U90926*, *Trbj2-3*). In contrast, Vgll3 and Taz sometimes co-induce the same genes (examples: *Gzmd, Unc5c*).

Click here to Download Table S5

Table S6: Genes deregulated by Vgll3 overexpression in mouse are also deregulated in man VGLL3 was knocked down (via siRNA) or overexpressed (via retroviral transduction) in human myoblasts. Cells were analysed in proliferating conditions (myoblasts) or after 2 days in differentiation medium (myocytes). mRNA was extracted and expression of the genes affected by Vgll3 in mouse (Fig 3) were analysed by RT-qPCR. Gene expression was normalized to control sample (si Control or RV control respectively) and fold change compared to control. Most of the genes deregulated by Vgll3 overexpression in mouse were validated in human myoblasts. However, overexpression of VGLL3 in proliferative myoblasts induces WWTR1, represses EGFR1 and does not affect FRMD6, WNT7B, PITX2/3 or IGFBP2-3.Data are presented as fold change, where a red colour indicates a significant difference ($p < 0.05$) between test sample (knockdown or overexpression of VGLL3) and control using a paired two-tailed t-test, where n=3 independent experiments.

Click here to Download Table S6

Journal of Cell Science • Supplementary information

Table S7: Vgll3 knockout does not affect fibre type distribution, fibre count, muscle weight, or cross-section area in hind limb muscles

(A) Total and relative fibre distribution in mouse soleus wildtype versus *Vgll3-/-* (mean values ± standard deviation).

(B) Cross-sectional area of muscle fibres in mouse soleus wildtype versus *Vgll3-/-* (mean values ± standard deviation).

(C) Absolute and relative weights of the hind limb muscles in relation to the total body weight.

Click here to Download Table S7

Table S8: Primary and secondary antibodies used

Antibody	Clone/Catalogue No.	Supplier	Concentration (IF)
Rabbit monoclonal anti-YAP	D8H1X	Cell Signaling	N/A
Mouse monoclonal anti-Myc	4A6	Merck	N/A
Mouse Monoclonal anti-Myogenin	F5D-s	DSHB	1/10
Mouse anti-MyHC	M20-c	DSHB	1/250
Mouse anti-β-Tubulin	E76-c	DSHB	1/300
Mouse anti-FLAG	F1804	Sigma	1/200
Chicken anti-GFP	ab13970	Abcam	1/2000
Rabbit anti-Laminin	L9393	Sigma	1/200
Mouse monoclonal anti MyHC1 Isotype IgG2b	BA.D5-c	DSHB	1/100
Mouse monoclonal anti-MyHC2a isotype IgG1	SC.71-c	DSHB	1/100
Mouse monoclonal anti-MyHC2b isotype IgM	BF.F3-c	DSHB	1/100
Secondary antibodies<br>Alexa fluor 350 Goat anti-Mouse IgG2b, Alexa fluor 488 Goat anti-Mouse IgG1, Alexa fluor 594 Goat anti-Mouse IgG1 and Alexa fluor 488 or 633 Goat anti Rabbit		Invitrogen	1/500

Journal of Cell Science • Supplementary information

Table S9: Primers used in the study

Gene	Forward primer (5'-3')	Reverse primer (5'-3')
Tbp	ATCCCAAGCGATTTGCTG	CCTGTGCACACCATTTTTCC
Vgll1	TTCAGGAGAACTGAAAGACGTG	GGGGGCATGCTCTTATTG
Vgll2	ACGCTTCCCAGCAAACAA	GGCTGGTCTTTCTCCTCCTC
Vgll3	GGATTCCTGCTCCCCAGT	TTGTCCTGATGCTGAAGACCT
Vgll4	TGTGAAAACGACCACGTCTC	GCAGTCTCCGTTGACAGTCTT
Pax7	CCGTGTTTCTCATGGTTGTG	GAGCACTCGGCTAATCGAAC
Myogenin	CTACAGGCCTTGCTCAGCTC	AGATTGTGGGCGTCTGTAGG
TBP	CGGCTGTTTAACTTCGCTTC	CACACGCCAAGAAACAGTGA
VGLL2	CTGTACCAGCAGCAAAGCAC	CATCGGGAAGGAGCAGTCT
VGLL3	TGGATGAACACTTCTCAAGAGC	GCTGGCTTGAGAGAGCTGAG
MYF5	CTATAGCCTGCCGGGACA	TGGACCAGACAGGACTGTTACAT
MYOGENIN	CCAGGGGTGCCCAGCGAATG	AGCCGTGAGCAGATGATCC
MyHC	AGCAGGAGGAGTACAAGAAG	CTTTGACCACCTTGGGCTTC
PITX2	CTGTGTGGACCAACCTTACG	CCGAAGCCATTCTTGCATA
PITX3	GAGTCTGCCTGTTGCAGGA	CAGCGTCTGACAGCGACA
WWTR1	ATTCGAATGCGCCAAGAG	AACTGGGGCAAGAGTCTCAG
AJUBA	TTTGTTTGCTGCTCTTGTGG	TGAAAACAGATAATCTTCCTCACAGT
AMOTL2	CAGCTTCAATGAGGGTCTGC	GCATGGAGCACCTTTAACCT
FRMD6	*CTGGTGCTCAAGACTTTCTCC*	*GGTTCCCAGCACTCCAAAG*
FSTL1	ACCCATCTTTCAACCCTCCT	GACACAGCGGTTACAGTCCA
EGFR	GTGGATGGCATTGGAATCA	CAAAGGTCATCAACTCCCAAA
WNT7B	TCATGAACCTGCATAACAATGAG	CCAGCAGGTTTTGGTGGT
FZD4	TTCACACCGCTCATCCAGTA	TGCACATTGGCACATAAACA
IGFBP2	CCAAGAAGCTGCGACCAC	GGGATGTGCAGGGAGTAGAG
IGFBP3	AACGCTAGTGCCGTCAGC	CGGTCTTCCTCCGACTCAC

Journal of Cell Science • Supplementary information
